# Antimicrobial Peptides Derived From Insects Offer a Novel Therapeutic Option to Combat Biofilm: A Review

**DOI:** 10.3389/fmicb.2021.661195

**Published:** 2021-06-10

**Authors:** Alaka Sahoo, Shasank Sekhar Swain, Ayusman Behera, Gunanidhi Sahoo, Pravati Kumari Mahapatra, Sujogya Kumar Panda

**Affiliations:** ^1^Department of Skin & VD, Institute of Medical Sciences, SUM Hospital, Siksha O Anusandhan University, Bhubaneswar, India; ^2^Division of Microbiology & NCDs, ICMR-Regional Medical Research Centre, Bhubaneswar, India; ^3^Department of Zoology, Maharaja Sriram Chandra Bhanja Deo University, Baripada, India; ^4^Department of Zoology, Utkal University, Vani Vihar, Bhubaneswar, India; ^5^Centre of Environment, Climate Change and Public Health, RUSA 2.0, Utkal University, Vani Vihar, Bhubaneswar, India

**Keywords:** antimicrobial peptide, biofilms, ESKAPE, insect, multidrug-resistant bacteria, molecular docking, anti-biofilm mechanism of action, therapeutic and prophylactic strategies

## Abstract

Biofilms form a complex layer with defined structures, that attach on biotic or abiotic surfaces, are tough to eradicate and tend to cause some resistance against most antibiotics. Several studies confirmed that biofilm-producing bacteria exhibit higher resistance compared to the planktonic form of the same species. Antibiotic resistance factors are well understood in planktonic bacteria which is not so in case of biofilm producing forms. This may be due to the lack of available drugs with known resistance mechanisms for biofilms. Existing antibiotics cannot eradicate most biofilms, especially of ESKAPE pathogens (*Enterococcus faecium*, *Staphylococcus aureus*, *Klebsiella pneumoniae*, *Acinetobacter baumannii*, *Pseudomonas aeruginosa*, and *Enterobacter* species). Insects produce complex and diverse set of chemicals for survival and defense. Antimicrobial peptides (AMPs), produced by most insects, generally have a broad spectrum of activity and the potential to bypass the resistance mechanisms of classical antibiotics. Besides, AMPs may well act synergistically with classical antibiotics for a double-pronged attack on infections. Thus, AMPs could be promising alternatives to overcome medically important biofilms, decrease the possibility of acquired resistance and treatment of multidrug-resistant pathogens including ESKAPE. The present review focuses on insect-derived AMPs with special reference to anti-biofilm-based strategies. It covers the AMP composition, pathways and mechanisms of action, the formation of biofilms, impact of biofilms on human diseases, current strategies as well as therapeutic options to combat biofilm with antimicrobial peptides from insects. In addition, the review also illustrates the importance of bioinformatics tools and molecular docking studies to boost the importance of select bioactive peptides those can be developed as drugs, as well as suggestions for further basic and clinical research.

## Introduction

A biofilm is a layer of polymeric organic matter to which microorganisms like fungi and/or bacteria, are attached in a sessile form. Biofilms are characterized by the presence of extracellular polymers, that create a visible slimy layer on a solid surface. The extracellular matrix: glycocalyx, can be made up of polysaccharides, proteins, glycoproteins, lipids, deoxyribonucleic acid, etc., which are collectively called extracellular polymeric substances (Costerton et al., 1987; Anwar et al., 1990; Matz et al., 2004). This glycocalyx provides a matrix for attachment of microbial cells and forms the internal architecture of the biofilm community. Biofilms have been defined as “a structured community of bacterial cells surrounded in a self-produced polymeric matrix and adherent to an inert or living surface” by Costerton et al. (1999) and “surface-associated microbial communities, surrounded by an extracellular polymeric substance matrix” by Hall-Stoodley and Stoodley (2009). A biofilm can also be defined as “a collective of microbial cells attached to a living or non-living surface, fixed within by a medium of extracellular polymeric substance matrix” (Hall-Stoodley et al., 2012). The most recent definitions treat them as “complex, sessile communities of microbes found attached to a surface in an extracellular matrix as aggregates” (Roy et al., 2018); and as a “well−organized structure formed by a bacterial community assemblage that is enclosed in a self−produced matrix in which bacterial cells communicate” (Armbruster and Parsek, 2018). We propose microbial biofilms as “communities of microbial cells with defined structures that attach on biotic or abiotic surfaces and are embedded in self-produced matrices consisting of extracellular polysaccharides, DNA and protein, with interspersed water channels.”

Peptides are biomolecules consisting of linear chains of amino acids that are found in almost every organism. Peptides can be generated as endogenous molecules for endocrine or neuronal signaling, or by degradation of proteins. The latter may have a positive impact on body functions as well as on health. These peptides perform major roles in the metabolic functions of living organisms, and (depending on their sequence) can have antimicrobial, anticancer, antidiabetic, anti-oxidative, immunomodulatory, etc., effects. Certain isolated bioactive peptides are used in the formulation or production of health-promoting food and food supplements, pharmaceuticals, cosmetics, or nutraceuticals. Most commonly, peptides can be generated from proteins by two methods, viz. chemical degradation (acid and alkali) (Andreu and Rivas, 1998), or enzymatic cleavage (Bongers and Heimer, 1994). With those methods, the original complex proteins from a plant or animal are broken down to yield peptides of 2-20 amino acid long residues. Nevertheless, the structure of peptides generated depends upon the native protein folding, degree of hydrolysis, enzyme specificity, and additional conditions of hydrolysis like temperature and time (Nehete et al., 2013). The amino acid sequence and composition are the primary characteristics of these peptides as these determine the specific bioactivity. Single peptides with a specific sequence can also be synthesized or produced by rDNA technology.

Under the above backdrop, the current review is planned to offer three aspects on the subject: (1) bacterial biofilm formation, (2) current therapeutic opportunities against biofilm producing bacteria, and (3) the probable role of insect-derived antimicrobial peptides in combating/reducing the biofilm formation. The first aspect includes bacterial biofilm composition, involvement of genetic virulence factors with reference to ESKAPE pathogens and its impact on human health and diseases. The second aspect discusses biofilm inhibition, dispersal, and eradication strategies along with the pathways and molecular mechanisms involved in biofilm formation. The third part of the review deals with AMPs from insect sources with some exclusive examples, the synergistic role of AMPs and antibiotics, and related *in vitro* studies and clinical trials. Besides, a section is added to emphasize the role of various bioinformatics tools and molecular modeling and docking analyses to accelerate the peptide-based drug discovery opportunities.

## Bacterial Biofilms and Their Formation

A biofilm, as a self-organized extra surface within the bacterial community, changes significantly the bacterial physiology in favor of exogenous stress tolerance and resistance to applied antibiotics or other biocides. Bacterial biofilms appear in both mono- and multilayers, depending on the attachment of the exopolysaccharide (EPS) matrix and the involvement of neighboring bacteria. The process of biofilm formation is complex and usually initiated by attachment of bacteria to a solid surface. Due to their hydrophobic nature, certain dissolved organic molecules accumulate on the solid: water interface, resulting in a film, and then they form small groups of bacteria, known as micro-colonies. EPS such as proteins, glycopeptides, glycolipids, lipopolysaccharides, and extracellular DNA accumulate in the attachment (Shirtliff et al., 2009). After completion of phase II irreversible microbial attachment, a mature biofilm forms, and the micro-colonies assume a distinct phenotype with a different gene expression than their planktonic counterpart (Stoodley et al., 2002). The process of differentiation can be activated by deposition of N-acyl homoserine lactones as a sensing molecule for cell to cell communication (Costerton et al., 1999). Biofilm formation is a good strategy for survival in a nutrient-poor environment suggesting starvation to favor biofilm formation. Besides, higher antibiotic resistance is observed when bacteria are grown or living under starvation. Biofilm development can be an adaptation of microorganisms to aggressive environments (De la Fuente-Núñez et al., 2013). Biofilm formation can happen on an assortment of surfaces, including living tissues and prosthetic implants (Donlan, 2002). About 99% of the microbial world exists in the form of biofilms containing a wide range of microorganisms (Garrett et al., 2008). For instance, over 500 types of microorganisms are found in biofilms in the oral cavity (Whittaker et al., 1996).

### Composition of Bacterial Biofilm

Microorganisms constitute 5-35% of the biofilm volume, the remaining part being extracellular matrix. The cellular matrix comprises proteins (e.g., fibrin), essential nutrients, and minerals from the surrounding environment. The extracellular matrix contains 1-2% polysaccharides (e.g., alginate), <1% DNA, <1% RNA, ions and 97% water. The flow of essential nutrients inside a biofilm is maintained through the water compartment. The EPS matrix (0.2-1.0 μm thick) strengthens the interaction of the microorganism’s and protects them from external factors like mechanical stress or antibiotics.

### Bacterial Genetic-Virulence Factors in Biofilm Formation, With Special Reference to ESKAPE

Biofilm-producing bacteria show higher (often 10-1000-fold) antibiotic tolerance/resistance to administered antibiotics in the biofilm compared to their planktonic state. Generally, antibiotic resistance factors such as mutations and efflux pumps are well understood in planktonic forms than in biofilm form (Munita and Arias, 2016). Thus, biofilm-associated antibiotic tolerance is assumed to involve alternative mechanisms. The ESKAPE (*E. faecalis*, *S. aureus*, *K. pneumoniae*, *A. baumannii*, *P. aeruginosa*, and *Enterobacter* species) pathogens are the leading biofilm-forming microorganisms causing nosocomial infections. Thus, understanding the unknown biofilm drug-resistance mechanisms is crucial for developing effective antimicrobial agents.

*Enterococcus faecalis* is the Gram-positive (Gm +ve) anaerobic bacterium among all *Enterococcus* species with resistance to antibiotics like ampicillin and vancomycin. The adhesion of bacterial cell to host tissues is the crucial step in biofilm production, which is mediated by enterococcal surface protein (Esp) involved in cell adherence, colonization, and persistence in the urinary tract, evasion of the immune system by aggregation protein (agg or asa1) and collagen-binding protein (aec) (Mohamed and Huang, 2007). The endocarditis antigens (efaAfs and efaAfm), endocarditis biofilm-associated pili (ebpABC), surface anchor protein, sortage (srt) and secretory antigens (salAB) like virulence factors are also involved in facilitating cell adhesion and biofilm formation (Chauang et al., 2000; Hashem et al., 2017). *Enterococcus* cells communicate through peptide pheromones cpd, cob, and ccf linking the receptor-donor pathways to transfer the biofilm regulator and promotor virulence genes. Mainly, the hemolytic exotoxins such as cytolysin A (cylA), autolysis (ata), hyaluronidase (hyl), gelatinase (GelE), and serine protease (sprE) are the most important virulence genes affecting host cells through regulating cell lysis and autolysis process (Seno et al., 2005; Thomas et al., 2008; Paganelli et al., 2013; Mottola et al., 2016; Naorem et al., 2020).

*Staphylococcus aureus* is another ubiquitous opportunistic Gm +ve pathogen, and the human nasal passage being the common route of infection in humans with a high risk of bloodstream infection and bacteremia in a later stage. Based on the flow-cell and microscopic studies, it is evident that initial *S. aureus* biofilm matrix formation occurs through polysaccharide intercellular adhesin (PIA) or polymeric N-acetyl-glucosamine (PNAG) which are responsible for synthesis, export, and modification of PIA as well as maintaining the structural integrity of the biofilm (Lauderdale et al., 2009; Brooks and Jefferson, 2014). Moreover, extracellular DNA or proteins can support biofilm formation in the absence of PIA (Rohde et al., 2007; Boles et al., 2010). Additionally, studies have also proved that a variety of proteins are available in *S. aureus*, viz. fibronectin−binding proteins A and B or fnbpAB (Cortés et al., 2011), fibrinogen−binding protein (fib) (Shannon and Flock, 2004), fibrinogen−binding protein clumping factors A and B or clfAB (O’Brien et al., 2002), biofilm−associated protein (bap) (Cucarella et al., 2001), collagen−binding protein (cna), serine−aspartate repeat proteins (Sdr) (Barbu et al., 2014), elastin−binding protein (Ebp) (Campoccia et al., 2009), and laminin−binding protein (eno) (Azara et al., 2017) that may regulate formation of biofilms in a strain- and environment-specific manner (Atshan et al., 2012; Tang et al., 2013; Serray et al., 2016).

*Klebsiella pneumoniae* is a Gm -ve ubiquitous bacterium, especially found in the intestinal tract of humans, but less common in the nasopharynx (Piperaki et al., 2017). In the pre-antibiotic period, *K. pneumoniae* was an important cause of pneumonia in the community, especially in alcoholics and diabetics while in past few decades it is established as the leading cause of diseases associated with health care in hospitals (Piperaki et al., 2017). Worldwide, *K. pneumoniae* infections rise in hospitals with severe antibiotic resistance making it difficult to treat patients, particularly immunocompromised individuals, and considered as the second most common cause of hospital-acquired Gm –ve infection (Candan and Aksöz, 2015; Paczosa and Mecsas, 2016). Schroll et al. (2010) found that type-3 fimbriae promote development of biofilm in catheter-associated infections caused by *K. pneumoniae*. Recently, Zheng et al. (2018) observed the biofilm formation to be more pronounced among magA (K1), aero+, rmpA+, rmpA2+, allS+, wcaG+, and iutA+ isolates than in isolates that were negative for these virulence factors; and concluded that presence of the wcaG virulence factor gene to be responsible for biofilm formation in *K. pneumoniae*.

*Acinetobacter baumannii*, a Gm -ve human pathogen (Family: *Moraxellaceae*, Class: *Proteobacteria* of *Eubacteria;* Evans et al., 2012) causes pneumonia, meningitis, bacteremia, wounds and soft-tissue infection, peritonitis and urinary tract infections (UTIs) (Dahdouh et al., 2017; Shirmohammadlou et al., 2018). Chronic infection, antimicrobial resistance and formation of biofilm in both biotic and abiotic surfaces are its important characteristics (Kongthai and Sitthisak, 2016). The rate of formation of biofilms is about 80-91% in case of *A. baumannii* (Sung, 2018). Different virulence factor proteins such as the outer membrane protein A (OmpA) with 38 kDa which play a crucial role in the attachment and attack to epithelial cells via contact with fibronectin; biofilm associated protein (Bap) (854 kDa), a cell surface protein responsible for cell to cell interactions and biofilm maturation; chaperon-usher pilus (Csu), are responsible for initiation of biofilm formation on abiotic surfaces (Dahdouh et al., 2017; Chapartegui-González et al., 2018; Ghasemi et al., 2018). In addition, EPS, two-component system (BfmS/BfmR), poly-β-(1,6)-N-acetyl glucosamine (PNAG) also participate in the formation of biofilm, quorum sensing system (Kongthai and Sitthisak, 2016; Ghasemi et al., 2018), virulence and antibiotic resistance (Amala Reena et al., 2017).

*Pseudomonas aeruginosa*, a rod-shaped Gm -ve bacteria, is also responsible to form biofilms (Tavares, 2000). Several virulence factors such as lipopolysaccharide (LPS), flagella, pili (type IV), exotoxin A, enzyme proteases, alginate, and QS, etc., are responsible for its pathogenicity. LPS perform a key role in the activation of the host’s innate (TLR4, NLRP1, NLRP2, and NLRP3) as well as adaptive immune responses. Eventually it aims to dysregulate the inflammation responses that compensates to high morbidity and mortality (Raetz and Whitfield, 2002). Type IV pili play a critical role in cell adhesion (Raetz and Whitfield, 2002). The T3SS (Type III secretion system) is another virulence factor which transports proteins from the cytoplasm of *P. aeruginosa* into the cytosol of host cells resulting phagocytosis by bacteria and damage to host tissues (Punsalang and Sawyer, 1973; Frank, 1997). The exotoxic A virulence factor is secreted by type II secretion mechanism, it secretes proteins in to the extracellular matrix conforming enzymes protease, lipase, phospholipase, and alkaline phosphatase which ultimately help the pilus-like apparatus (Passador and Iglewski, 1994).

The family *Enterobacteriaceae* is a complex group of Gm -ve bacteria basically found in the intestinal tract and urinary tract and most common cause of UTI and lower respiratory tract infections (Peleg and Hooper, 2010; Magiorakos et al., 2017). The virulence factors include different adhesins, hemolysin production and serum resistance, etc. aids to form biofilms in the human intestine that can affect the colon, and have a significant effect on the functioning of the intestinal microbiome and its interaction with the gut (Rossi et al., 2018).

### Impact of Biofilm on Human Health

The concept of biofilm is not new in medical microbiology. Antoni van Leeuwenhoek, as early as 1683, observed and described biofilms through his primitive microscope using matter from his own teeth. However, the concept of biofilm was not an essential concept in medical microbiology until early 1970s. Recognition of persistent infection and aggregates of bacteria in cystic fibrosis patients by Høiby (2017), and later evidence of biofilm involvement in pathogenesis in chronic infection and antibiotic treatment failure familiarized the concept in medical microbiology (Bjarnsholt, 2013). Today, it is an emerging field of research in mechanism of antibiotic resistance and antibacterial drug development.

**FIGURE 1:**
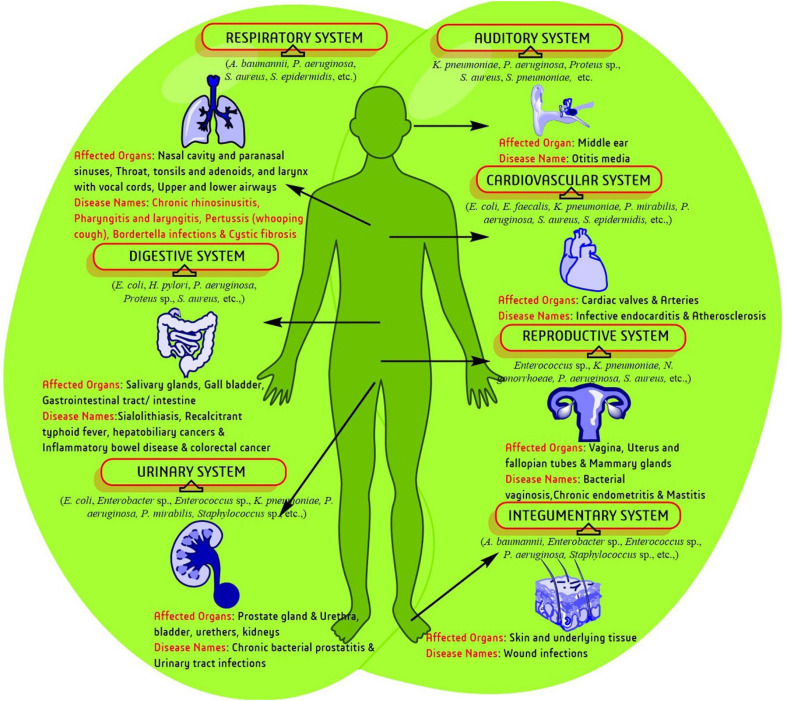
A schematic representation of biofilm-associated human diseases and affected biological systems/organs.

Biofilm production is a part of bacterial survival mechanisms and is associated with several health complications (Figure 1). Currently, both communicable and non-communicable diseases are associated with infection, where biofilm enhances complicacy of disorder in most of the cases. The genetic disorder cystic fibrosis (CF) was the first recognized biofilm infection model and so far, the most thoroughly studied one. CF generally affects the respiratory and digestive systems and are characterized through the production of viscid mucus and chronic infection. Lungs infecting and biofilm predicting strains like *S. aureus, H. influenzae*, and *P. aeruginosa* lead to mortality of CF patients as age advances (Lyczak et al., 2002). Biofilm like-structure have primarily been observed in lung lavage samples and apical surface of respiratory epithelia in CF patients which stimulate epithelia to increase the secretion of inflammation medicating factors. Biofilm producing pathogens were continuously isolated from other respiratory disorders such as chronic rhinosinusitis, pharyngitis or sore throat, and pertussis or whooping cough (Vestby et al., 2020).

Bacteria are continuously presented in urinary system disorders like bacterial prostatitis (BP) and UTI. Acute BP infection harbors several pathogenic bacteria such as, *Escherichia coli*, *Proteus mirabilis*, *P. aeruginosa*, *Klebsiella* sp., *Enterobacteriaceae* and *E. faecalis* which are associated with infertility, bladder infection, prostatic abscesses, urosepsis, kidney damage, bacteremia, septicemia, and death. Biofilm producing *E. coli* also frequently occur in BP and UTI and enhance the complications by reducing the potency of antibiotics administered (Flores-Mireles et al., 2015). The human integumentary system is mostly associated with and effected by pathogenic bacteria. Damaged living tissue attract various bacterial species. Sometimes healing is disrupted, and wound tissues acquire severe infection by planktonic bacteria or biofilm producing bacteria. Wound infection in immuno-compromised, geriatric, and diabetic cases is more complicated (Bjarnsholt et al., 2011). Biofilm has been assumed to be the underlying cause for transformation of wounds into chronic stages. Several studies have confirmed that biofilm or biofilm producing pathogens are directly associated with the failure of antibiotics and delay in optimal wound healing/management (Zhao et al., 2013).

Bacterial vaginosis (BV) is the most common genital tract infection in women throughout reproductive years during which several anaerobic bacteria such as *Gardnerella vaginalis*, and *Atopobium vagnae* decreases the number of protective lactobacilli. Generally, the vaginal population of *G. vaginalis* of a healthy woman has less chance of causing BV. However, several transcriptome studies proposed that, *G. vaginalis* can result different phenotypes of the pathogen through large changes in gene expression (Kumar et al., 2011). Simultaneously, environmental pressure and ecological disturbances in the vaginal niche produces biofilms influenced by other bacterial species leading to pathogenesis. Similarly, chronic endometritis complications are also affected by biofilm producing *E. faecalis*, *E. coli*, *G. vaginalis*, *K. pneumoniae*, *P. aeruginosa*, *Staphylococcus* sp., and *Streptococcus* sp. Acute otitis media (OM), one of the most common pediatric inflammation agents in the middle ear cavity of <5-year aged children, is also caused by biofilm development that leads to high-risk pathogenesis through colonization of the otopathogenic bacteria, *S. pneumoniae*.

Typhoid fever, the most acute food borne illness generally caused by *Salmonella enterica*, is associated with several complications and even death if untreated. Several studies in a murine gall stone model with *S. typhimurium* identified biofilm formation in the gall bladder in chronic typhoid carrier states (Gonzalez-Escobedo et al., 2011). Antibiotics, those generally effective against the acute infection, are ineffective against the biofilm associated chronic colonization of the gall bladder. Other digestive system-associated disorders like ulcerative colitis and Crohn’s disease, an inflammatory bowel disease (IBD) lead to chronic inflammation of the digestive tract with some common symptoms like pain, diarrhea, weight loss, fatigue, etc. *Bacteriodes fragilis*, *Enterobacteriaceae*, and *E. coli* produce biofilms in epithelium site which stimulate an inflammatory response over failure of maintaining the integrity of the mucosal barrier resulting in a reduced ability to clear the infection.

Other specific clinical observations have confirmed that bacterial biofilms were pledged in every patient with clinical post-operative infections (∼75%) and recurrent sialadenitis (a digestive disorder that forms stones in salivary gland)/pus drainage. The presence of bacterial biofilms may enhance the severity of sialadenitis. Isolated microorganism (staphylococci, streptococci, enterococci, etc.) from infective endocarditis (a cardiovascular disorder) also produce biofilms, which is involved in physical disruption of valve function and overcome antibiotic therapy by bloodstream infection. Recently, biofilm producing bacteria have also reported in atherosclerotic arteries through fluorescence microscopy and fluorescence in situ hybridization (FISH) (Vestby et al., 2020).

## Current Therapeutic Practices Against Biofilm Producing Bacteria

Biofilm is the bacterial community’s self-motived mechanism for pathogenesis and, alternatively, associated with antibiotic/antibacterial resistance/tolerance. Recently, several methods have been examined to tackle biofilm or biofilm-producing bacteria through innovative techniques such as non-coating, surface coating or individual/synergistic antibiotics, anticancer drugs, natural products, and peptides in application-specific manner (Pletzer and Hancock, 2016; Li et al., 2018; Reen et al., 2018; Verderosa et al., 2019b). Concomitantly, several experiments with different model systems to prevent biofilm formation are going on. The overall strategy may be divided into three parts as described below:

### Biofilm Inhibition Strategy

Several target specific strategies have been used to control/inhibit bacterial biofilm development. As bacterial adhesion promotes mature biofilm formation, preventing bacterial attachment or bacterial adhesion is an ideal strategy. Development of medical devices, biomaterials or coating could alter the surface morphology of target tissues to avoid an extension unfavorable to bacteria (Bazaka et al., 2012; Li et al., 2017). Arciola et al. (2012) have shown an effective method for preventing biofilm-related infections/complications associated with orthopedic implants. Overall, this method is more suitable for large-scale surface modification to prevent biofilm formation (Bazaka et al., 2012; Campoccia et al., 2013). Similarly, using a small therapeutic inhibitor/agent is another approach used to prevent the formation of biofilm. The biofilm inhibitors are often employed to passivate the medical biomaterial/devise (Boase et al., 2018). A wide variety of biofilm inhibitors of bromo-pyrrole, furanone, imidazole, indole, phenol, etc., class of compounds have been reported (Simões et al., 2010; Worthington et al., 2012; Rabin et al., 2015).

### Biofilm Dispersal Strategy

Biofilm dispersal agents mostly target the biofilm activated/regulated bacterial biochemical pathways such as QS, c-di-GMP, and sRNAs pathways. Inhibiting the function of enzymes involved in biofilm matrix formation is one such approach (Kaplan, 2010; McDougald et al., 2012; Fleming and Rumbaugh, 2017). These disperser cells are more suitable in antimicrobial treatment than biofilm-residing cells and currently, this approach becomes an intense area aiming to develop promising dispersal agents (Fleming and Rumbaugh, 2017; Roy et al., 2018). Briefly, alginate lyase of *P. aeruginosa*, a surface-protein-releasing enzyme of *S. mutans*, thermonuclease of *S. aureus*, LapG protease of *P. putida*, hemagglutinin protease of *Vibrio cholerae*, endo-β-1,4-mannanase of *Xanthomonas campestris* are some well-characterized target enzymes for biofilm dispersal strategy (Kaplan, 2010). However, this treatment method becomes problematic if the disperser cells are not treated or translocated into new areas, which may spread the infection like the initial stage. Therefore, in most cases, a potent dispersal agent is a co-administrated/synergistic approach with an antimicrobial agent to get a promising result (Marvasi et al., 2014; Reffuveille et al., 2015; Roizman et al., 2017).

### Biofilm Eradication Strategy

Currently, the development of novel antimicrobial agents to eradicate biofilm is an emerging area of research. Till date, several promising agents have already been developed/proposed including antimicrobial peptides or AMPs like LL-37, oritavancin, novispirin G10, etc., quaternary ammonium compounds or QACs like tris-QAC-10, XF-70, XF-73, etc. (Jennings et al., 2014; Forman et al., 2016); antimicrobial lipids like glycerol monolaurate, docosahexaenoic acid, etc. (Schlievert and Peterson, 2012; Sun et al., 2016); nitric oxide releasing antibiotics or nitro-oxide functionalized antibiotics, like cephalosporin-3’-diazeniumdiolate, ciprofloxacin-nitroxides-27, poly (amidoamine) dendrimer, etc. (Barraud et al., 2009; Worley et al., 2015; Verderosa et al., 2016, 2019a); redox-active secondary metabolites or phenazines and quinolones (phenazine-14, bromophenazine-8, halogenated quinoline-3, etc.) (Cezairliyan et al., 2013; Garrison et al., 2015; Basak et al., 2016; Huigens et al., 2019). Many of them were proved against biofilm producing ESKAPE pathogens. Overall, the use of AMPs as an alternative to antibiotics and particularly biofilm has received significant attention over a couple of years.

### Pathways and Molecular Mechanisms Involved in Biofilm Formation

**FIGURE 2:**
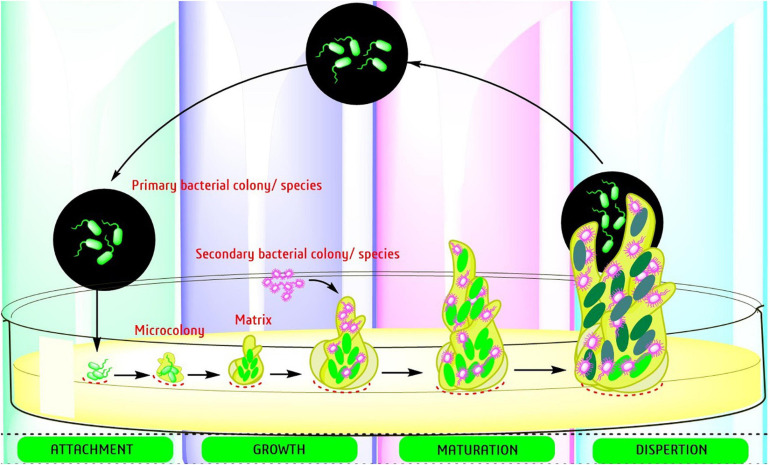
Step by step processes toward the development of bacterial biofilms.

The development of a biofilm occurs in four distinct stages: (a) attachment to a surface, (b) binding to the surface through adhesins followed by both a reversible and irreversible process of extension, (c) development of micro-colonies, and (d) maturation of biofilm architecture (Figure 2). Bacterial biofilm formation is a highly regulated process that occurs through bacterial quorum sensing (QS), bis-(3′-5′)-cyclic di-guanosine monophosphate (c-di-GMP) and small RNAs (sRNAs) pathways.

Quorum sensing is a unique signaling pathway in the bacterial community that maintains communication through a small “autoinducer” molecule that leads to biofilm formation. When a surficial density of bacteria is present and the autoinducers’ concentration reaches a threshold level, bacteria start to activate certain target genes. Overall, QS controls 10% of the bacterial genome and plays a crucial role in the formation and dispersal of biofilms through the proposed signaling. The QS-system is not involved in the biofilm’s initial attachment or growth stage but is the main pathway for biofilm dispersal.

The second primary biofilm regulator pathway is the c-di-GMP network the most complex secondary signaling bacterial system that varies between species. After binding to various cellular receptors, c-di-GMP regulates bacterial transcription, enzyme activity, and larger cellular structures via QS signaling. Overall, c-di-GMP plays a decisive role in the switch between planktonic and biofilm formation, as well as biofilm structure development through the synthesis of exopolysaccharides, adhesive pili, secretion of extracellular DNA, along with regulating cell death and survival. Lastly, the non-coding sRNA molecules actively participate in bacterial post-transcriptional gene regulation, metabolic processing, stress adaptation and microbial pathogenesis (Mandin and Gottesman, 2010; Michaux et al., 2014). Overall, sRNA plays an influential role in the biofilm life cycle.

**FIGURE 3:**
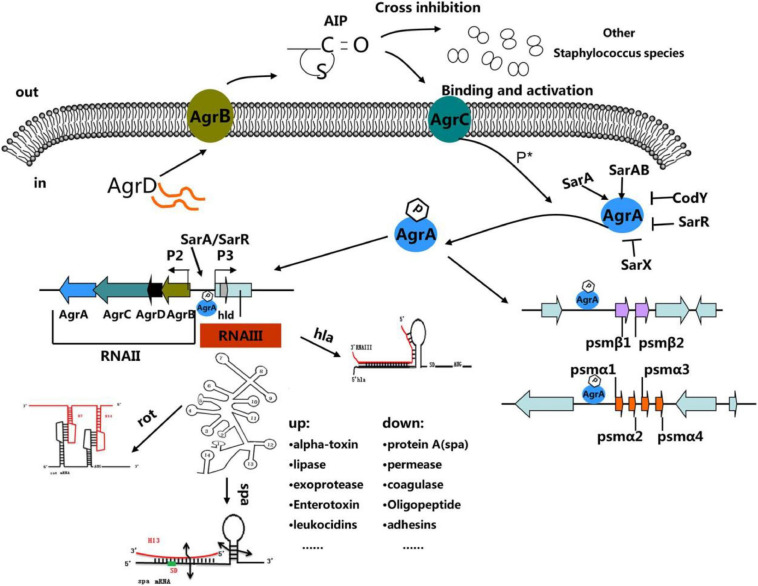
“The *Staphylococcus* quorum-sensing system. The agr locus is composed of divergent transcripts designated RNAII and RNAIII, driven by promoters P2 and P3, respectively. The AIP signal is produced from the AgrD precursor, while the membrane-localized enzyme AgrB participates in the maturation and export of the AIP. At a critical threshold concentration, AIP activates the two-component signal transduction system, AgrC–AgrA, and causes the phosphorylation of AgrA. Once phosphorylated, AgrA binds to the P2 and P3 promoter regions, as well as promoters PSM-a and PSM-b, resulting in agr system transcription. RNAIII encodes the delta-toxin encoding gene hld, and 14 stem-loop motifs. These domains regulate the expression of numerous virulence factors. Other regulators (such as SarA, SrrAB, SarR, and SarX) can enhance or inhibit agr activity (Tan et al., 2018).”

Tan et al. (2018), published a review interpreting the “molecular mechanisms underlying agr quorum sensing and the regulation of agr expression.” They, deemed that agr system is as an attractive therapeutic target for controlling *S. aureus* by blocking or interfering with the agr system. For detail pathways, see Figure 3.

Recently, Yan and Wu (2019) reviewed the transcriptomic data and validated the possibility to reverse the biofilm formation in *P. aeruginosa* through QS. The QS system of *P. aeruginosa* constitutes 3 systems including its own specific QS system (PQS). For more details, see Figure 2, Yan and Wu (2019). Besides, Qvortrup et al. (2019), in a recent review, described the “anti-biofilm agents developed on the basis of mechanistic understanding of biofilm formation” and highlighted the process of biofilm formation and the molecular mechanism used as targets for the development of anti-biofilm chemicals with special reference to *A. baumannii, E. coli*, and *P. aeruginosa.*

Biofilm formation by *P. aeruginosa* can be initiated via the adhesive action of a number of components such as “flagella (O’Toole and Kolter, 1998), type IV pili (O’Toole and Kolter, 1998; Déziel et al., 2001; Chiang and Burrows, 2003), cup fimbria (Vallet et al., 2001), extracellular DNA (Whitchurch et al., 2002), and Psl polysaccharide (Ma et al., 2006).” Most of the biofilm matrix components such as polysaccharide, exopolysaccharide, alginate, CdrA, type IV pili, and Cup fimbriae are positively controlled by c-di-GMP (Fazli et al., 2014), which is a negative controller of motility of *P. aeruginosa* (Simm et al., 2004). It is witnessed that biofilm infection with *P. aeruginosa* can be treated by a reduction of the bacterial c-di-GMP content (Christensen et al., 2013). Quorum sensing (QS) plays an important role in the formation and persistence of *P. aeruginosa* biofilms. Although several compounds are controlled by QS, extracellular DNA that contributes toward antimicrobial resistance and the stability of biofilms is an important factor (Allesen-Holm et al., 2006; Chiang et al., 2013). The other responsible molecules is the rhamnolipid in the development of biofilm formation by phagocytizing immune cells (Pamp and Tolker-Nielsen, 2007).

In the case of *E. coli*, formation of biofilm is regulated by several adhesins and extracellular matrix components. The proteinaceous curli fibers are a major component required for the initial attachment to the host cells for biofilm formation (Olsén et al., 1989; Nasr et al., 1996; Prigent-Combaret et al., 2000; Chapman et al., 2002; Serra et al., 2013). The other important components are type 1 and P pili (Pratt and Kolter, 1998; Schembri and Klemm, 2001; Niba et al., 2008). It is evidenced that absence of FimH molecules significantly reduces adhesion capability both *in vitro* and *in vivo* (Langermann et al., 1997; Mulvey et al., 1998). The other factor is P pili (contain PapA subunits), that anchors the adhesin PapG (Gong and Makowski, 1992; Bullitt and Makowski, 1995), enables to bind *E. coli* to host epithelial cells (Busch et al., 2015). Beside the proteinaceous component, it is also evidenced that the exopolysaccharides, poly-GlcNAc (PGA), and colanic acid (Danese et al., 2000; Wang et al., 2004; Serra et al., 2013; Subashchandrabose et al., 2013; Besharova et al., 2016) and extracellular DNA are the key components of *E. coli* biofilm formation (Devaraj et al., 2015). In *E. coli*. Like *P. aeruginosa*, PGA and curli fimbria are also positively regulated by c-di-GMP.

The mechanisms of biofilm formation in *A. baumannii* is not well studied. Nevertheless, a few adhesins and extracellular components are detected for their role in biofilm formation. Csu pili and the OmpA are the outer membrane protein (Dorsey et al., 2002; Tomaras et al., 2003; Gaddy et al., 2009) which binds to epithelial cells to form the biofilm. BAP, another important protein, is responsible for cell-cell adhesion and in maintaining the structure of mature biofilms (Loehfelm et al., 2008). Furthermore, this bacterium can produce the exopolysaccharides alginate and poly-β-1,6-N-acetylglucosamine (PNAG) which can serve as an important constituent of the biofilm matrix (Lee et al., 2008; Choi et al., 2009). So far, the role of c-di-GMP signaling in *A. baumannii*, biofilm formation has not been documented. While QS has been proven to regulate the formation of biofilm (Anbazhagan et al., 2012), *A. baumannii* have an “AHL-based QS system with AbaI functioning as the AHL synthase and AbaR functioning and the AHL receptor.” Niu et al. (2008) observed that an AbaI mutant (could not produce AHL), have imperfections in the late stage of biofilm formation. Addition of AHL developed an increased expression of Csu pili, as well as stimulation of biofilm formation (Luo et al., 2015).

## Antimicrobial Peptides (AMPs)

Antimicrobial peptides are a widespread feature of the innate immune systems, a principal defense system, present in almost all living organisms ranging from fungi to higher plants and animals. So, both eukaryotic and prokaryotic cells produce AMPs naturally as part of their immune/immune system (Rossi et al., 2008). Generally, a peptide’s primary role is to kill the invading pathogens (bacterial, fungal, viral, parasitic, etc.) through modulating the innate immune response of the host. However, the activities vary in different host systems depending on the organism and its location in that organism. Bacteria were among the first source of AMPs (called bacteriocins), which could be a new therapeutic source for various human ailments. Bacterial AMPs do not defend against infection by other species of bacteria; they kill other species (target) of bacteria as a source of nutrients or to decrease competition for nutrients. Some AMPs are narrow spectrum, most of the broad-spectrum activities target various bacterial enzymes, pathways, or structures (like lipid bilayers).

Distinctive characteristics of most AMPs are their small size containing 15 to 30 amino acids along with positively charged ones, and that they target the cell membrane (Rossi et al., 2008; Melo et al., 2009). As a result, positively charged peptides are attracted to cell membranes compared to poorly (negatively) charged ones by bacteria and biofilm sites. AMPs lead to increased antimicrobial activity in bacteria that are active and slow-growing in biofilms (Ma et al., 2012; Xu et al., 2014; Tiwari et al., 2015) and easily kill them (Jorge et al., 2012). However, in low-intensity environments, AMPs can be bacteriostatic (Beloin et al., 2014). AMPs are also classified depending on their secondary structure in liquid media (Dalton and March, 1998; Stoica et al., 2017). Some are mainly beta-pleated-sheet structures whereas others are primarily alpha-helical. In both the cases, cysteines form an intramolecular disulphide bridge, that stabilizes the structure, and helps AMPs cross the cell membrane (Whittaker et al., 1996; Garrett et al., 2008). Cell adhesion is favored due to hydrophobic interactions of AMPs (Costerton et al., 1999; Reffuveille et al., 2014; De La Fuente-Núñez et al., 2016). The antibacterial activity depends on the balance between the charge density, the hydrophobic character, and the length of the polypeptide chain (Stewart, 1996; Mah and O’Toole, 2001; Arciola et al., 2012). Increasing the number of basic amino acids or altering their configuration in the peptide chain can affect the secondary structure of AMPs, and thus their antibacterial activity. Insects are known to be highly resistant to bacterial infections. They can produce many proteins and peptides as the first line of defense against microbial infection (De La Fuente-Núñez et al., 2016).

### Antibacterial Peptides From Insect Source

**FIGURE 4:**
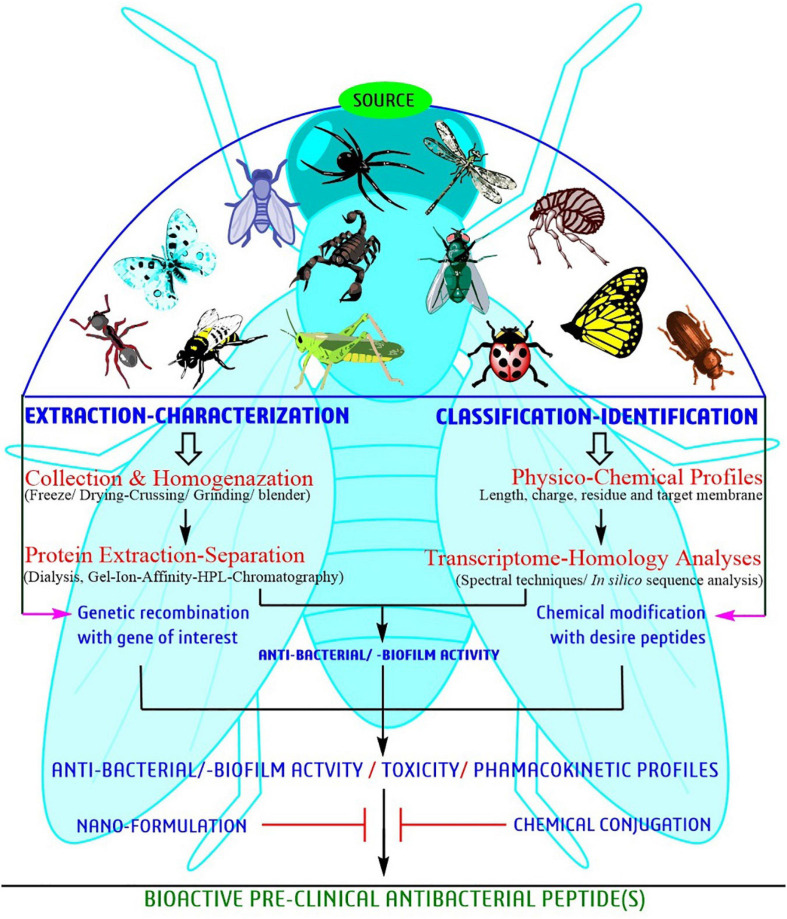
A systematic approach of bioactive peptide identification and characterization toward development of potent AMPs against biofilm-producing pathogens.

Since the time AMPs were first discovered forty years back, researchers have attempted to relate amino acid sequences for antibacterial activities in order to get better peptides (Figure 4). Bioassay-guided purification seems to be the best strategy. However, information on bioassay-guided purification is rare. Figure 4 provides a schematic presentation of a systematic approach for identification and characterization of bioactive peptides for developing potent antimicrobial peptides. The regular methodology is to substitute amino acids in the arrangement to control cationic charge and hydrophobicity and obtaining about 5-10 peptides that are tasted for antimicrobial activity. For the parent sequencing, the derived peptides record moderate function against antimicrobial activity (Fjell et al., 2012; Haney et al., 2012; Kim H. et al., 2016; Agrawal et al., 2018), or may be having lower toxic effect, and ∼1–4 of these peptides may be concentrated further to determine their mechanism of action. A large number of sequences are registered in the databases. For example, >3000 sequence are present in AMP Database (Wang et al., 2016) and >17000 sequences in DRAMP database (Fan et al., 2016).

The development of high-throughput techniques, like next-generation sequencing and transcriptome analysis, is helping to find new antimicrobial peptides in different organisms. The process involves total RNA extraction, construction of RNA library, sequencing, and *de novo* assembling of transcriptome (Haas and Zody, 2010). In addition, different strategies of *in silico* analysis are being used to identify peptides of therapeutic interest, predict their three dimensional structures and enrich the search for antimicrobial peptides based on physicochemical properties and nucleotide sequence similarity (Lata et al., 2007; Amaral et al., 2012; Slavokhotova et al., 2015). Chemical synthesis of the identified/predicted AMPs can be carried out through solid-phase chemical synthesis and purified via RP-HPLC and obtained with differential purity levels. Mass spectroscopic technique is useful to determine the molecular mass of the molecules. Such molecules can be tested against selected microbes for antimicrobial activity. Before being finalized to be used as a drug, the molecules can be tested for their cytotoxic and human erythrocyte hemolytic activity. However, the molecular mechanism of these peptides is largely not known till date.

About 103 antimicrobial peptides isolated from insect sources are listed in Supplementary Table 1. The majority of AMPs are derived from insects like *Acalolepta luxuriosa, Apis mellifera*, *Bombyx mori, Galleria mellonella, Heterometrus spinifer, Holotrichia diomphalia, Hyalophora cecropia, Oxysternon conspicillatum*, *Pandinus imperator*, and *Sarcophaga peregrine*. These antimicrobial compounds are found to be effective against a wide range of bacteria both from Gm -ve and Gm +ve, including MDR strain (Supplementary Table 1). The MIC in most insect peptides is quite potent (0.02 to 20 μg/mL). Many of them are known to prevent the formation of biofilms in ESKAPE bacteria (Table 1). Sojka et al. (2016) isolated defencin-1 (derived from defensin) from *A. melifera* with strong antibacterial activity against both planktonic and biofilm cells of the bacterial pathogens such as *S. aureus, S. agalactiae, P. aeruginosa* and *E. faecalis* (MIC = 0.009-0.09 μM). Another broad-spectrum antimicrobial peptide coprisin isolated from *Copris tripartitus* has antibiofilm property against a wide range of pathogenic bacteria such as *E. faecium, S. aureus, E. coli, S. mutans, P. aeruginosa* (MIC = 1.7-3.4 μM) (Hwang et al., 2009). Melittin, isolated from *A. mellifera* has broad spectrum activity against Gm -ve, *P. aeruginosa, E. coli*, and *K. pneumoniae* (MIC = 0.0001-0.0008 μM) (Dosler et al., 2016; Memariani et al., 2019). Mastoparan-1 is a narrow spectrum AMP isolated from *Polybia paulista* and effective against methicillin-resistant *S. aureus* (MRSA) (Memariani et al., 2018). Table 1 provides information on a list of potent AMPs reported to inhibit biofilm.

**TABLE 1:** Experimentally demonstrated AMPs derivatives from insects having antibiofilm properties.

Peptide	Source (Taxonomic name/common name)	Process of synthesis	Purification method	Characterization	Active against MDR pathogen	MIC in μ M	References
*A3 (derived from AamAP1)	*Androctonus amoeruxi* (African fat tail scorpion)	SPM using Fmoc Chemistry	RPHPLC	ESI-MS	*S. aureus*	25	Almaaytah et al., 2018
BmKn-22 (derived from BmKn-2)	*Mesobuthus martensii* (Manchurian scorpion)	*ChinaPeptides Co., Ltd. (Shanghai, China)*	NM	NM	*P. aeruginosa*	200-800	Teerapo et al., 2019
Cecropin-A	*Galleria mellonella* (Greater wax moth)	SPM using Fmoc Chemistry	RPHPLC	ESI-MS	*E. coli* (UPEC)	11.86	Kalsy et al., 2020
Coprisin	*Copris tripartitus* (Dung beetle)	NM	NM	MALDI-TOF MS	*E. faecium, S. aureus, E. coli, S. mutans, P. aeruginosa*	1.7-3.4	Hwang et al., 2013
*Defensin-1 (derived from defensin)	*Apis melifera* (Western honey bee or European honey bee)	NM	(Ni-NTA) agarose affinity chromatography	NM	*S. aureus, S. agalactiae, P. aeruginos, E. faecalis*	0.009-0.09	Sojka et al., 2016
Mastoparan-1	*Polybia paulista* (Neotropical social wasp)	SPM using Fmoc Chemistry	RPHPLC	ESI-MS	Methicillin-resistant *S. aureus* (MRSA)	0.001-0.019	Memariani et al., 2019
Mastoparan-C	*Vespa crabro* (European hornet)	NM	RPHPLC	ESI-MS	*P. aeruginosa, S. aureus*	32	Chen et al., 2018
Mauriporin	*Androctonus mauritanicus* (Fat tailed scorpion)	SPM using Fmoc Chemistry	RPHPLC	ESI-MS	Methicillin-resistant *S. aureus* (MRSA)	5 -10	Almaaytah et al., 2014
Melittin	*Apis mellifera* (Western honey bee or European honey bee)	NM	NM	NM	*P. aeruginosa, E. coli, K. pneumoniae*	0.0001-0.0008	Dosler et al., 2016; Memariani et al., 2019
*Pro10-1D (derived from protaetiamycine)	*Protaetia brevitarsis* (White-spotted flower chafer beetle)	NM	NM	NM	*E. coli, A. baumannii*, other MDR bacteria	4	Krishnan et al., 2020

*^∗^,synthetic form of insect peptide, NM, not mentioned; SPM, solid phase methods, Fmoc, 9-fluorenylmethoxycarbonyl; RPHPLC, reverse phase high performance liquid chromatography; ESI-MS, electrospray ionization mass spectrometry; MALDI-TOF MS, matrix-assisted laser desorption/ionization-time of flight mass spectrometry; MIC, minimum inhibitory concentration.*

Oxysterlins 1, 2, and 3, isolated from the dung beetles *O. conspicillatum* (Toro Segovia et al., 2017), similar to Cecropin A, Aedesin, Lucilin and HKABF, were found selective for Gm –ve bacteria and efficiently kill multidrug resistant (MDR) strains, including *E*. *coli* ESBL, *E*. *cloacae*, *S. typhi*, and *E*. *coli* with MIC values between 3.12 and 50 μg/mL. Cecropins were first isolated from the silk moth *H. cecropia*. Insect cecropins also have other names such as bactericidin, lepidopteran, sarcotoxin etc. “Cecropins can lyse bacterial cellular membranes and can also inhibit proline uptake as well as cause leaky membranes (Moore et al., 1996; Bechinger and Lohner, 2006).” Cecropin B is found to have the strongest antibacterial activity (Srisailam et al., 2000) and proved to decrease the load of *E. coli* in a rat model (Giacometti et al., 2001). A recombinant cecropin D was found to be active against both Gm + and Gm -ve bacteria (Guo et al., 2012). Most probably the C-terminal lysine residue is responsible to increase the antibacterial activity due to activated phosphorylation (Park et al., 2013).

### Mechanisms of Antibiofilm Peptides With Special Reference to ESKAPE Pathogens

Insects are capable to produce variety of antimicrobial proteins and peptides most of which are smaller in size and contain cationic groups. Generally, antimicrobial peptides are categorized according to their structure such as α-helical, β-sheet, loop, or extended constructions; however, some does not fit into any specific class due to the presence of both α-helical and β-sheet domains. Thus, the peptide structures can be observed through membrane interactions (Jenssen et al., 2006). For example, indolicidin like neutrophil peptides from bovine is unstructured in an aqueous medium but become boat-like after interaction with the membrane mimicry surface of sodium dodecyl sulfate and dodecyl phosphocholine. The antibacterial mechanism of each peptide depends on its character/composition and physiological conditions. A peptide contains many positively charged residues that enable them to interact electrostatically with negatively charged cell surface molecules of surface peptidoglycan. Overall, isolated short cationic amphiphilic host-defense peptides are responsible for antibacterial activity through direct cell killing and immunomodulatory action (Zasloff, 2019; Mukhopadhyay et al., 2020). In general, peptides are highly effective against Gm -ve bacteria than Gm +ve bacteria due to differences in cell wall composition. Additionally, non-specific inhibition mechanisms target membrane, intracellular biomolecules, and oxidative pathways (Wimley and Hristova, 2011; Band and Weiss, 2014). The therapeutic potential of peptide antibiotic drugs lies in their ability to kill bacterial cells effectively without exhibiting significant cytotoxicity toward mammalian cells. Overall, the activity and mode of peptides are variable due to their structure like β-hairpin or loops, β-sheet and amphipathic α-helical constituents (Gomes et al., 2018; Hollmann et al., 2018).

Several models have been projected to elucidate the bacterial cell membrane disruption out of which, the Barrel-stave, Carpet model and Toroidal-pore are the most accepted models (Zasloff, 2019). Mechanically, peptides disrupt/kill/inhibit the bacterial growth through some essential steps such as attraction, attachment, insertion, and inhibition of bacterial biofilm formation. In the attachment stage, peptides penetrate the entire depth of bacterial surface polysaccharide and further join with the lipopolysaccharides in Gm -ve bacteria and teichoic/lipoteichoic acid in Gm +ve bacteria (Figure 4, left side). The right side of Figure 4 describe a computational model with a molecular docking approach graphically targeting LuxR genetic factor of *S. aureus* with insect-derived antibiofilm peptide Pro10-1D. Several computational methods like molecular docking and dynamic simulation are the most widely used, cost-effective programs to elucidate several unknown features, mechanisms, and potency targeting individual biofilm-associated targets.

### Some Exclusive Examples of Antibiofilm Peptides Derived From Insects

Basically, four groups of AMPs are found in insects based on their structure and amino acid composition. They include proline rich peptides (e.g., drosocin, apidaecin, and lebocin), α-helical peptides (e.g., moricin and cecropin), cysteine rich peptides (e.g., defensin and drosomycin), and glycine-rich proteins (e.g., attacin and gloverin) (Otvos, 2000; Bulet and Stocklin, 2005). The major components of innate immunity in numerous groups of organisms including insects are cysteine-rich peptides (Pushpanathan et al., 2013; Slavokhotova et al., 2017) and are known for their molecular action to inhibit biofilms. In addition to these several new class of insect peptides is still isolated from several insects; diptericins, drosomycin metchnikowin and ponericins are most investigated antibacterial insect peptides. On the other hand, most insect peptides proved antibacterial activity in planktonic compared to biofilm. Overall, the glycine and proline-rich peptides are significantly active against Gm -ve bacteria (Wu et al., 2018).

Defensins are very small (<4 kDa) antibacterial peptides containing three intramolecular disulphide bridges by the help of six cysteine residue and are found in all living organisms. They are classified in to three families based on their structural characteristics, such as classical defensins, β-defensins and defensins of insects (Ganz and Lehrer, 1994). Vertebrate defensins in innate immunity have attracted many workers (Ding et al., 2009; Lehrer and Lu, 2012; Jarczak et al., 2013; Zhu et al., 2013; Wilson et al., 2013; Zhao and Lu, 2014). Insect defensins contain a cationic group of 34–51 peptide residues with six conserved cysteines. It basically inhibits Gm +ve bacteria, including *S. aureus.* Some insect defensins also perform their biological activity (Maget-Dana and Ptak, 1997). The AMPs lucifensin isolated from *Lucilia sericata* contain 44 amino acid residue (Čeřovský et al., 2010). However, the molecular mechanism of cysteine rich peptides is not well established against biofilms.

**FIGURE 5:**
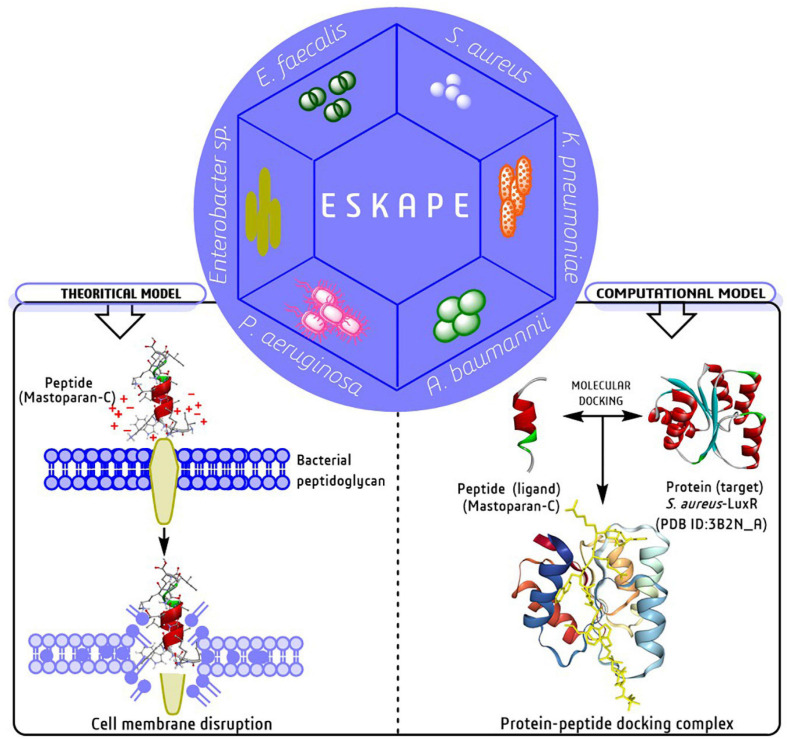
Graphical representation of membrane disruption mechanism of peptides against ESKAPE pathogens in both theoretical and computational models (Chen et al., 2018; Silveira et al., 2021).

The other important classes of insect peptides are α-helical peptides (e.g., moricin and cecropin). Cecropins were first isolated from the pupae of the cecropia moth *H. cecropia*, whence the term cecropin was derived. Basically, different groups of cecropins are found such as cecropins A, B, C, D, E, and F among which Cecropin D with 37 amino acids is the major cecropin. They function against both Gm -ve and Gm +ve bacteria as well as fungi (Kockum et al., 1984; Tryselius et al., 1992; Moore et al., 1996; DeLucca et al., 1997; Cavallarin et al., 1998; Ekengren and Hultmark, 1999; Vizioli et al., 2000). Most cases of cecropins at the C-terminal end are altered to amide, and amidation is essential for the interaction of cecropins with liposomes, and hence the antimicrobial property (Li et al., 1988). The basic molecular mechanism of cecropins is described recently by Mukherjee on *E. coli* biofilm (Figure 5; Mukherjee, 2020).

Glycine-rich proteins e.g., attacin and gloverin are the other important insect-derived peptides. Attacin (MW-20-23 kDa), isolated and purified from the hemolymph of the immunized bacteria *H. cecropia* and its isoforms, can be divided into two groups: the basic attacins (A–D) and acidic attacins (E and F) (Hultmark et al., 1983). Both are similar in amino acid sequence but acidic attacin contains higher proportion of Asp residues compared to basic attacin. Besides, both are encoded by two different genes (Kockum et al., 1984; Sun et al., 1991). Attacins are synthesized as inactive pre-proproteins with a signal peptide, a pro-peptide (P domain), and an N-terminal attacin domain followed by two glycine rich domains (G1 and G2 domains) (Sun et al., 1991; Hedengren et al., 2000). Attacin-coleoptericin is a hybrid protein with greater antibacterial activity against *E. coli, Burkholderia glumae*, and *B. subtilis* related to either attacin or coleoptericin alone (Lee et al., 2013). But leucine-rich attacins do not exhibit antimicrobial activity. Attacins mostly act by blocking the synthesis of the major outer membrane proteins of Gm -ve bacteria, as a result disturbing the integrity of the cell wall and causing the bacteria to grow in long chains (Carlsson et al., 1998). Attacin causes increased permeability of outer membrane of *E. coli* and inhibits synthesis of outer-membrane proteins at the transcriptional level without entering the inner membrane or cytoplasm (Carlsson et al., 1998). Biofilm-associated bacteria are more resistant to antibiotics than live (planktonic) cells. Despite the availability of several reports on the antibacterial property against Gm –ve bacteria with isolation of attacin from different insect species, no such data is available on biofilm which should attract priority. The antibiofilm mechanism of action, assay and techniques associated with antimicrobial peptides from insect sources are summarized in Table 2.

**TABLE 2:** The antibiofilm mechanism of action, assay and techniques for insect-derived AMPs.

Peptide	Assay/Technique	Mechanism of action	References
A3	MBEC; MBC	Membrane disruption	Almaaytah et al., 2018
BmKn-22	QS (swarming motility assay, protease and pyoverdin assay)	Cell signaling	Teerapo et al., 2019
Cecropin-A	MBIC	Membrane disruption	Kalsy et al., 2020
Coprisin	MBIC	Membrane disruption	Hwang et al., 2013
* Defensin-1	MBC, MBEC	Membrane disruption	Sojka et al., 2016
Mastoparan-1	Solid-surface assay on MRSA	Cell surface and subsequent destabilization of bacterial membrane	Memariani et al., 2018
Mastoparan-C	MBIC, MBEC; Membrane permeability assay	Membrane disruption	Chen et al., 2018
Mauriporin	MBEC	Membrane disruption	Almaaytah et al., 2014
Melittin	MBIC	Membrane disruption	Dosler et al., 2016; Memariani et al., 2019
Pro10-1D	MBIC, microscopic observation	Membrane disruption	Krishnan et al., 2020

MBEC, minimal biofilm eradication concentration; MBIC, minimal biofilm inhibitory concentration; MBC, minimum bactericidal concentration; QS, quorum sensing. *, synthetic derivative.

At present, most insect peptides can be synthesized in laboratory conditions and identified the potency through computational screening at an early stage. Indeed, the advanced *in silico* next-generation sequencing, transcriptome profiling, *de novo* assembly directed toward locating signature bioactive peptides (Kim I.-W. et al., 2016; Prajapati et al., 2020). Briefly, *in silico* analysis, able to find out the vital function with their encoded genetic factors from the large sequence (Toro Segovia et al., 2017). On the other hand, the modification of natural peptides in laboratory conditions also provides massive success in achieving the desired goal of improving activity and pharmacokinetics profiles. For example, several glycosylated and unglycosylated analog of the insect peptide apidaecin and drosocin were proposed by Gobbo et al. (2002) where synthesized unglycosylated containing intrachain disulfide bond have more potential activity than glycosylated derivatives (Gobbo et al., 2002). Similarly, A3 (AamAP1), BmKn-22 (BmKn-2), defensin-1, (defensin), Pro10-1D (protaetiamycine), several other synthetic derivatives, namely, LL1037, LL7-31 (LL-37), 1018 (bactenecin), AS10 (CRAMP), battacin (lipopeptides), BMAP27-melittin (melittin), CAMA (cecropin A and melittin A), NRC-16 (pleurocidin), P10 (P60.4Ac), P318 (CRAMP), are some improvised insect peptides toward control of biofilm (De La Fuente-Núñez et al., 2016; Almaaytah et al., 2018; Krishnan et al., 2020).

### Antimicrobial Peptides: Synergy Studies With Antibiotics

Biologically, the synergistic approach of non-homologous insect peptide(s) and conventional antibiotics/antibacterial agents showed potential against resistance pathogens. Overall, the interaction of peptide and membrane is directly promotional to antibiofilm/antibacterial activity (for overall inhibition mechanisms refer Figure 4). Mechanically, a peptide kills bacteria through different routes, including disruption by electrostatic or hydrophobic interaction, interference of bacterial metabolism, targeting cytoplasmic components, etc. Currently, four models such as, barrel-stave, carpet, toroidal-pore, and disordered toroidal-pore are used to predict the modes of action of peptides and all models required a threshold concentration to conduct antibacterial activity (Hollmann et al., 2018; Wu et al., 2018; Manniello et al., 2021; Silveira et al., 2021). In general, the activity and mode of peptides are variable due to their structure (Gomes et al., 2018; Hollmann et al., 2018). For example, the potential cecropin A or CecA peptide isolated from *G. mellonella* recorded an antibiofilm activity; but, when applied synergistically with nalidixic acid, showed significant antibiofilm activity at lower concentration against uropathogenic-cum-biofilm producing *E. coli* (Kalsy et al., 2020).

De La Fuente-Núñez et al. (2012) have shown that AMP 1037 stimulates cell proliferation of *P. aeruginosa* PA2204 but does not affect the motility and biofilm formation (De La Fuente-Núñez et al., 2012). Antimicrobial peptides NA-CATH: ATRA1-ATRA1, a synthetic cathelicidin, inhibited *S. aureus* biofilm form, and peptide LL-37 regulated *P. aeruginosa* biofilm formation when used at levels below MIC (Overhage et al., 2008; Dean et al., 2011). These AMPs inhibit the expression of coded proteins in the genes involved in the formation of biofilm. In *P. aeruginosa*, genes contain type IV pili code, rhamnolipid synthesis, quorum sensing, and flagella assembly (Overhage et al., 2008). However, a few AMPs have specific antimicrobial properties; for example, lactoferrin chelates iron and inhibits biofilm formation by *P. aeruginosa*. Binding AMPs to extracellular DNA may improve the detection of biofilms (Das et al., 2010).

**FIGURE 6:**
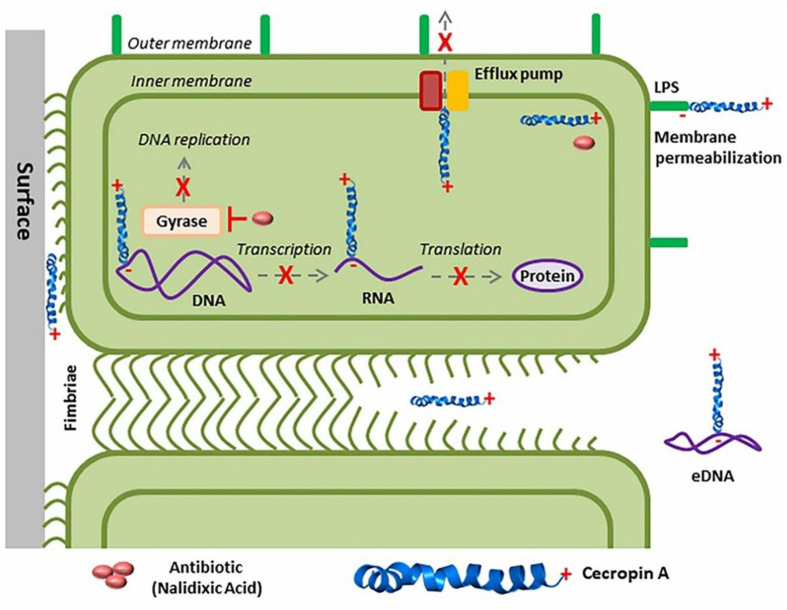
Multi-target mechanism of cecropin A (CecA) action in uropathogenic *Escherichia coli* (UPEC) biofilms. CecA interacts with LPS to permeabilize bacterial membrane enhancing the diffusion of nalidixic acid (NAL) into the cell. CecA and NLA then bind to different molecular targets in bacteria to disrupt UPEC biofilms (Adopted from https://naturemicrobiologycommunity.nature.com/posts/59576-in-search-of-new-anti-biofilm-agents-from-insects, Mukherjee, 2020).

The *S. aureus* biofilm formation regulatory system (GraRS) perform a crucial role in resisting microorganisms in AMPs (Herbert et al., 2007). This resistance was inverted when AMPs were added in mixtures with other antimicrobial compounds. AMPs from a variety of sources, when combined with standard prescribed antibiotics, effectively prevent the formation of biofilm by *P. aeruginosa* (Eckert et al., 2006; Minardi et al., 2007; Herrmann et al., 2010; Hirt and Gorr, 2013; Dosler and Karaaslan, 2014). Combination of A3 (isolated from *Androctonus amoerux*) with conventional antibiotics performed a synergistic mode of action when compared with the natural scorpion venom peptide. The combination of A3 with conventional antibiotics may be used as a possible new treatment strategy against MDR and biofilm forming bacteria (Almaaytah et al., 2018). Combination of BmKn–22 peptide with azithromycin also reduces *P. aeruginosa* biofilms (Teerapo et al., 2019). Kalsy et al. (2020) have synergistically reported that insect antimicrobial peptide cecropin A (CecA) can reduce planktonic and sessile biofilm-forming UPEC cells, either alone or in combination with the antibiotic nalidixic acid (NAL) (Figure 6). Coprisin also performed antibiofilm activity alone and in mixtures with antibiotics (Hwang et al., 2013).

Akbari et al. (2019) observed interesting synergistic effects of peptide melittin when combined with existing antibiotic doripenem against *A. baumannii* (61.5 fold reduction on MIC) as well as against *P. aeruginosa* (31.5 fold reduction on MIC). Same group also demonstrated two new melittin-derived peptides MDP1 and MDP2 with potent antibacterial activity against MDR *S. aureus* and *E. coli* (Akbari et al., 2018). Melittin also inhibits MRSA strains (Choi et al., 2015) and later proved to eradicate MRSA-infected mice (Moreno and Giralt, 2015). Another recent finding with AMP against MDR *S. aureus* is clavaspirin (Lee et al., 2018). Indeed, few synergistic effects of AMPs along with conventional antibiotics were demonstrated (Akbari et al., 2019) but quantitative methods are rarely used, or *in vivo* validation is not performed. Another synergetic study is by Wu et al. (2017) with AMP DP7 on MDRs (*S. aureus, E. coli*) using quantitative polymerase chain reaction.

### *In vivo* Studies Related to Antimicrobial Peptide Research and Insect

*In vivo* studies has shown the effect of interactions on the biofilms of MRSA in which nisin was mixed with daptomycin/ciprofloxacin; indolicidin and teicoplanin, cecropin (1-7), melittin A (2-9), and amide (CAMA) with ciprofloxacin (Mataraci and Dosler, 2012; Dosler and Mataraci, 2013). The combination of cationic peptide IB-367 and LZD in antibiotic lock technology eliminated *S. aureus* biofilms in CVCs (Ghiselli et al., 2007). Significant reduction of biofilm-associated *S. aureus* in vascular grafts was observed when sub-MIC levels of vancomycin was combined with the lipopeptides Pal-Lys-Lys-NH2 and Pal-Lys-Lys (54). Some AMPs have a wide range of antibiotic activity, such as peptide 1018, which is blocked or degraded by guanosine [pentaphosphate (p) ppGpp] and is essential for biofilm formation. In the lower extremities, peptide 1018 inhibited the formation of biofilm but eliminated pre-packaged biofilms when applied at high concentrations (De La Fuente-Núñez et al., 2014). AMPs combined with generic antibiotics may be a better alternative than antibiotics alone. The interaction of AMPs with antibiotics against biofilm viruses should draw the attention of researchers to study the mechanical properties of these compounds.

## Clinical Trials Related to Insect Derived Antimicrobial Peptides

Out of the 208 drugs approved during the last five-years (2015-2019) (FDA-drug approval records), 15 (approximately 7%) are peptide-based drugs (De La Torre and Albericio, 2020). As a result, the peptide-based drug market/business crossed one billion USD. Newer strategy and agents are urgently needed to counter-attack the biofilm-producing and multidrug-resistant pathogenic bacteria. At the same time, the potency, non-specific antimicrobial inhibition mechanism and other advantages over conventional antibiotics are rapidly gaining attention as an intellectual source toward the development of peptide-based antibacterial drugs (Browne et al., 2020; Magana et al., 2020). Bacitracin, daptomycin, colistin, gramicidin D, oritavancin, polymyxin B, teicoplanin, telavancin, vancomycin, etc., are some well-known peptide based-successive and revolutionized antibacterial (Browne et al., 2020). Similarly, many antimicrobial peptides are entering clinical trials with several positive responses and withdrawn from different clinical trial stages due to toxicity, lack of *in vivo* stability pharmacokinetic properties (Butler et al., 2013; Koo and Seo, 2019). Herein, relevant clinical status reports of ten antibacterial peptide based drug information such as origin, mechanism of action, route of administration, resisted clinical trials number are tabulated, particularly those currently presented in clinical-II onward (Koo and Seo, 2019; Browne et al., 2020).

Briefly, as per potency, several drugs are now in a different stage of clinical validation (Table 3). As we know, clinical proof is a long-term process that focuses on the lead candidates’ dose and adverse effects. MU1140, D2A21, Arenicin (AP139), AP138, Novarifyn, hLF1-11, Wap-8294A2 (10, lotilibcin) like peptide-based antibacterial drug candidates are entering in the clinical trial-1 study. Similarly, EA-230, CZEN-002, Delmitide, Ghrelin, C16G2, DPK-060, PAC113, LTX-109, OP-145, LL-37, Novexatin like candidates in trail-II and D2A21, SGX942, PXL01, POL7080, POL7080, Ramoplanin are in trail-III stage. Overall, most of the drugs failed in phase-II and III due to their inability to reach the required clinical endpoints. For example, omiganan, pexiganan and surotomycin like lead drugs are withdrawn in clinical trial-III due to lesser potency. Similarly, iseganian, talactoferrin (for mortality) and murepavadin (significantly for higher renal toxicity), were stopped/to be withdrawn due to serious adverse effects in comparison with control (Divyashree et al., 2019; Koo and Seo, 2019; Theuretzbacher et al., 2020). Additionally, NVB-302, XMP-629, Neuprex, and Friulimicin B like candidate drugs failed to achieve the required clinical features. Thus, clinical acceptance is always the last part of drug development where isolation/identification of such potential peptide candidates plays a significant role in initiating that drug development strategy.

**TABLE 3:** Some potent exclusive antibacterial/anti-infective peptide-based therapeutical regimens (which are exited in clinical trials in phase II onward/not terminated in the clinical phase).

Name (Sponsor)	Original source (species)	Application	Mechanisms	Route of administration	Number of participants, study region	Clinical trial number (NCT*)
Brilacidin/PMX-30063 (Innovation Pharmaceuticals)	Host defense peptides/defensin (Human)	Acute bacterial skin infections, biofilm infection, asthma, acne, COPD	Membrane permeabilization, inhibition of PDE4, modulate cAMP pathway	Intravenous/Topical	61, Unstated states	02324335
DPK-060 (DermaGen & Pergamum AB)	Kininogen (Human)	Acute external otitis, eczematous lesions infection	Bacterial membrane disruption	Topical	69, Sweden	01447017
Histatin/P-113 (Demgen)	Histatin (Human)	Chronic *P. aeruginosa* infections, gingivitis, and periodontal diseases	Disruption of pathogens plasma membrane, intracellular components	Topical	223, United States and North Carolina	00659971
Lytixar/LTX-109 (Lytix Biopharma)	Synthetic anti-microbial peptidomimetic	Atopic dermatitis, mild eczema, nasal and MRSA associated infection	Control bacterial infection through bacterial membrane disruption/permeabilization	Topical	24, Hungary	01223222
Murepavadin/POL7080 (Polyphor Ltd.)	Protegrin I (Pig/Porcine)	Ventilator-associated *P. aeruginosa* pneumonia/lower respiratory infection	Outer membrane lipopolysaccharide transport protein D	Intravenous	25, Greece and Spain	02096328
OP-145 (OctoPlus/Aleš Rozman/Calhoun Vision, Inc.)	Cathelicidin LL-37 (Human)	Chronic otitis media, biofilm associated infection, wound healing	Bacterial toxins neutralisation	Topical	84, Slovenia; 600, United States	01366261 01496066
Pexiganan/MSI-78/Locilex (Dipexium Pharmaceuticals)	Magainin (Frog)	Infected diabetic foot ulcers	Bacterial membrane disruption	Topical	189, United states; 200, United states	01590758 01594762
p2TA/AB103 (Atox Bio Ltd)	Recombinant from chorionic gonadotropin hormone (Human)	Necrotising soft tissue infection	Immunomodulation	Intravenous	290, United States and France	02469857
Surotomycin/CB-183, 315 (Merck Sharp & Dohme Corp, Cubist Pharmaceuticals LLC)	Daptomycin (Actinobacteria)	Clostridium difficile-associated diarrhoea	Membrane depolarisation	Oral	608 606 30 40	01598311 01597505 02835118 02835105
						

*, synthetic derivative.

Strategically, several specific chemical modifications/synthetic conjugations are also available to boost the clinical success rate with withdrawn lead candidates and improved antibiofilm activity against different pathogenic bacteria (De La Fuente-Núñez et al., 2016). A3 (AamAP1), BmKn-22 (BmKn-2), defensin-1, (defensin), Pro10-1D (protaetiamycine), several other synthetic derivatives namely, LL1037, LL7-31 (LL-37), 1018 (bactenecin), AS10 (CRAMP), battacin (lipopeptides), BMAP27-melittin (melittin), CAMA (cecropin A and melittin A), NRC-16 (pleurocidin), P10 (P60.4Ac), P318 (CRAMP), are some chemical modification attempts toward improving the activity against biofilm than their parental form (De La Fuente-Núñez et al., 2016; Almaaytah et al., 2018; Krishnan et al., 2020). Similarly, nano-synthesis techniques like nanoparticle, nano-formulation, liposome, PLGA nanoparticle drug delivery also significantly help overcome the lack of physiochemical properties and pharmacokinetic profiles of peptides (Biswaro et al., 2018; Dostert et al., 2019; Galdiero et al., 2019). No such combination is available for the insect. Documentation of more scientific literature, critical discussion, and assessment will help peptide-based antibacterial/antibiofilm future drug development strategy.

## Bioinformatics Tools to Accelerate the Peptide-Based Drug Discovery

Currently, *in silico* drug discovery approach or computational intelligence platform-based drug discovery plays a crucial role both in academia as well as in industry by locating possible active molecules from a bunch of desired molecules, predicts the structure and function of the active molecule with homologous/similar candidates information, able to predict toxicity-pharmacokinetic profiles based on chemical composition, mechanism of inhibition targeting any particular disease associated targets/enzyme, etc., (Malathi and Ramaiah, 2018; Duarte et al., 2019; Swain et al., 2020). Similarly, various tools, databases and software’s of bioinformatics can also help speed up the peptide-based research through analysis within a limited time and resources (Gupta et al., 2016; Agrawal et al., 2018; Câmara et al., 2020).

The universal National Center for Biotechnology Information (NCBI)^1^, and Universal Protein Resource or UniPort^2^, protein data bank or PDB^3^, with some specific sources such as data repository of antimicrobial peptides or DRAMP^4^, update linking antimicrobial peptides or LAMP2^5^. Yet another database of antimicrobial peptides or YADAMP^6^, database of antimicrobial activity and structure prediction of DBAASP^7^, collection of antimicrobial peptides or CAMPR_3_^8^, classification of antimicrobial peptides or ClassAMP^9^, structure database of bioactive peptides or StraPep^10^, ligand-protein binding database or BioLip^11^ are the most important open access resources able to guide any peptide associated research from prediction to submission. To date >200 peptide base databases are available for any kind of reference and study.

From the history of antibacterial drug discovery, validation of any kind of potential antibacterial agents (herein peptides) are facing a complex validation procedure or filtrate through different ideal drug parameters. Physiochemical properties play a crucial role in selecting the most potent antibacterial-cum-antibiofilm peptides at the first stage of clinical trials. On the other hand, the revolution in bioinformatics tools toward predicting ideal drug candidates directly reduces the time, resource, and cost in current drug discovery. Briefly, in the preclinical trial, most of the compounds showed the most potent activity during *in vitro* testing; however, most candidates do not contain physiochemical and are withdrawn later in different clinical trial stages. For example, the predicted hydropathicity index or GRAVY represents the hydrophobicity of the respect insect peptide. According to ExPASy, positive GRAVY values indicate hydrophobic and negative values indicated the hydrophilic value as a crucial parameter for clinical validation. The isoelectric point (pI)/IEP is the value at which the overall net surface charge is widely used in proteomics and peptide-based drug discovery. For a peptide, pI values mostly depend on the acid dissociation constant (*pKa*) of the ionizable groups of charged amino acids glutamate (δ-carboxyl group), aspartate (ß-carboxyl group), cysteine (thiol group), tyrosine (phenol group), histidine (imidazole side chains), lysine (ε-ammonium group), and arginine (guanidinium group). Overall, a peptide’s net charge is strongly related to pH and greatly influenced in druggable candidate selection. Similarly, molecular weight, half-life period, etc., are smaller parameters with significant influence in most potential peptide selection. Bioinformatics tools predict the possible values without any expensive experiments through an extensive training dataset. Thus, different bioinformatics not recommended but guided toward peptide-based drug discovery.

Similarly, the universal bioinformatics tools such as, protein homology/analogy recognition engine2.0 or Phyre2^12^, protein secondary structure prediction or PSIPRED^13^, iterative threading assembly refinement or I-TASSER^14^, basic local alignment search tool or BLAST^15^, physicochemical analysis tool, ProtParam^16^, pharmacokinetic-toxicity property prediction tool, SwissADME^17^ with some specific tools namely, improved prediction of antimicrobial peptide or iAMPpre^18^, biofilm-active peptides or BaAMPs^19^, collection of antimicrobial peptides signature or CAMPSign^20^, the *de novo* approach peptide structure prediction tool, PEP-FOLD^21^ and for protein-peptide/protein-protein docking tools, HPEPDOCK^22^, HDOCK^23^, ClusPro^24^, and PeptiDock^25^ helps significantly in peptide research. Advanced artificial intelligence techniques and huge biological information always provide ideal opportunities to make some desired/objective-oriented tools for guidance at the early stage of peptide drug discovery by predicting several anonymous druggable information.

### Physicochemical Property and Homology/Phylogenetic Analysis

Bioactive peptide identification in current antibacterial drug discovery era is one of the most interesting research area (Jakubczyk et al., 2020). The amino acid composition of a peptide directly influences drug-likeliness or drug suitability characteristics, including absorption, distribution, metabolism, excretion, and toxicity (ADMET) properties. Moreover, an ideal isoelectric point, hydrophobicity, lipophilicity, half-life period, the amino acid composition, mass, etc., collectively known as physicochemical properties, is an essential feature for a lead bioactive therapeutic peptide (Boone et al., 2018; Zafar et al., 2020). The above properties are always estimated by chromatographic techniques; however, this technique is expensive and time-consuming to calculate protein fragments one by one in a considerable amount of data set. On the other hand, most bioactive peptides are withdrawn from the clinical study due to unfavorable physicochemical properties. Several computational based tools have been developed taking a massive number of training data sets as a possible solution; thus, anyone can check/predict the above properties freely before synthesis/derivatization or an exponential study. For example, the universal tool, ProtParam was used to estimate the physicochemical properties for selected antibiofilm insect-derived peptides (Table 4) and this information guides/useful for peptide-based antibacterial/antibiofilm drug-development research.

**TABLE 4:** Predicted physico-chemical properties for selective antibiofilm insect peptides using bioinformatics tool ProtParam.

Sl. No.	Total length	MW (Da)	pI value	NC	NP	Instability index	Aliphatic index	Half-life (hour)	GRAVY score
1	18	1981.4	11.7	0	3	7.95	141.11	1.1	1.233
2	11	1188.5	11.0	0	2	1.37	160.00	1.1	1.218
3	39	4215.01	10.21	3	9	21.53	95.13	1.3	-0.177
4	45	4728.5	8.67	2	5	26.59	86.67	100	0.193
5	95	10717.4	6.27	14	13	35.04	77.05	30	-0.081
6	14	1655.01	8.50	2	3	33.89	153.57	20	0.064
7	14	1507.97	10.30	0	3	10.91	209.29	5	1.279
8	73	8417.06	10.39	8	17	48.43	102.88	30	-0.256
9	70	7584.86	4.69	9	6	51.70	106.00	30	0.239
10	12	1676.99	12.48	0	4	144.93	105.83	1	-1.050

*GRAVY, Grand average of hydropathicity; Half-life value was estimated in mammalian reticulocytes, *in vitro*; NC, Total number of negatively charged residues (Asp+Glu); NP, Total number of positively charged residues (Arg+Lys); pI, theoretical isoelectric point prediction.*

Similarly, PepCalc.com^26^, PepDraw^27^, peptide property calculator^28^, protein descriptor calculator^29^, peptide analyzing tool^30^, etc., are several freely accessible tools that are available to predict the physicochemical properties of any desired peptide.

Homologous/structural similarity compound search is another high-through approach in ongoing drug discovery toward discovering more bioactive compounds in a minimum time and resource (Kanduc, 2012; Zheng et al., 2019). For example, if someone finds an active peptide from an insect or any other source and validated it to have potent antibiofilm property, then next homologous search is more appropriate to search structurally related/identical peptide from different databases than comparison with any physical documentation. The well-known protein-BLAST or BLASTp^31^, Phyre2, PSIPRED, SPIDER^32^, HHpred^33^, etc., are different bioinformatics tools that give homologous information to accelerate the peptide-based drug discovery. On the other hand, phylogenetic search helps give the proper identification or even source of an unknown peptide. Generally, a homologous peptide having a common origin is always present in the same clade or branch of a phylogenetic tree. The clustal omega^34^ and the molecular evolutionary genetics analysis or MEGA^35^ are the most widely used open-source bioinformatics tools for phylogenetic tree analysis. Overall, this homologous-cum-phylogenetic search is commonly used in genomics and proteomics analysis. Thus, bioinformatics is always trying to produce more relevant tools/software to reduce computational and experimental peptide research gaps.

### Molecular Modeling and Docking Analysis

Molecular modeling is a technique used to generate a three-dimensional (3D) structure of any protein, peptide or DNA structure using two different approaches, namely homology modeling with known template and *de novo*/physical design without any template (Nikolaev et al., 2018). The 3D structure of any peptide or protein always gives some relevant information on the active amino acid geometry/active site of a disease associated enzyme for drug development modules.

**FIGURE 7:**
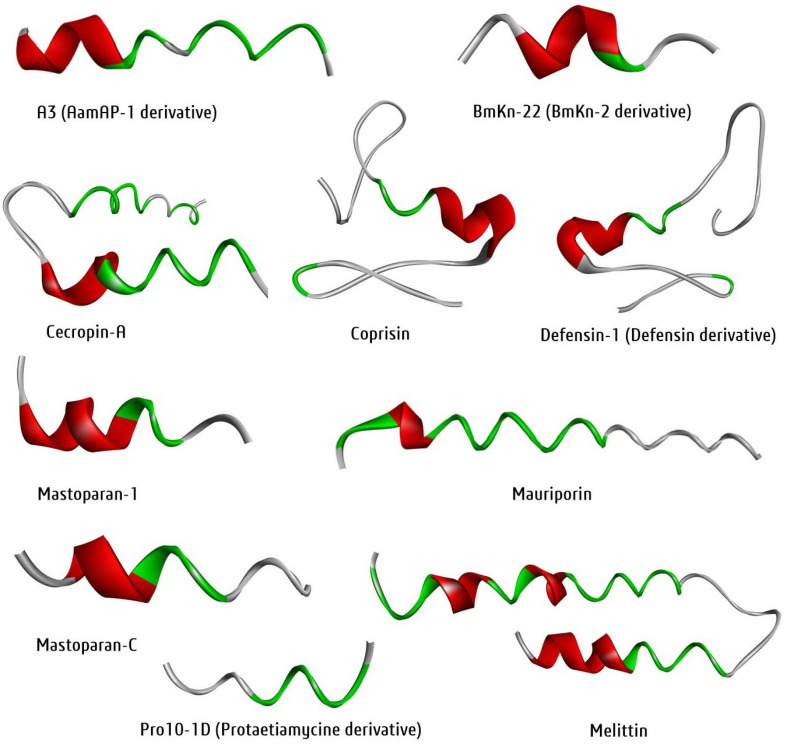
Three-dimensional structure of newly generated ten active antibiofilm peptide derivatives using the PEP-FOLD3 tool. The BIOVIA-Discovery studio visualizer software was used for 3D-visualization in secondary structure format with clean geometry. The backbone protein portion is more fat than other regions, where red color indicated helices regions, gray color indicated coil regions and green color indicated the turn regions of the protein structure.

Protein data bank is one of the largest and open-source 3D-structural database of peptides, proteins, DNAs, and nucleic acids generated through NMR, X-ray or cryo-EM. However, this technique is costly and limiting. At the same time, bioinformatics tools can generate the 3D structure followed by cost-effective homology and *de nove* modelling approach to overcome the structural complexity in drug discovery. Notably, the homology approach tools, SWISS-MODEL^36^, CPHmodels 3.2^37^, and *de novo* approach tools, Phyre2, I-TASSER, PEP-FOLD3^38^ are most prevalent for 3D protein/peptide structure prediction. For example, the 3D structure of the ten selected antibiofilm insect-derived peptides were generated with the PEP-FOLD3 tool and visualized using the software BIOVIA-Discovery Studio Visualizer (Figure 7). Thus, molecular modeling an essential tool in a systematic and cost-effective platform for peptides. Similarly, molecular docking is another essential tool for computer-aided drug discovery platforms (Swain et al., 2018).

**FIGURE 8:**
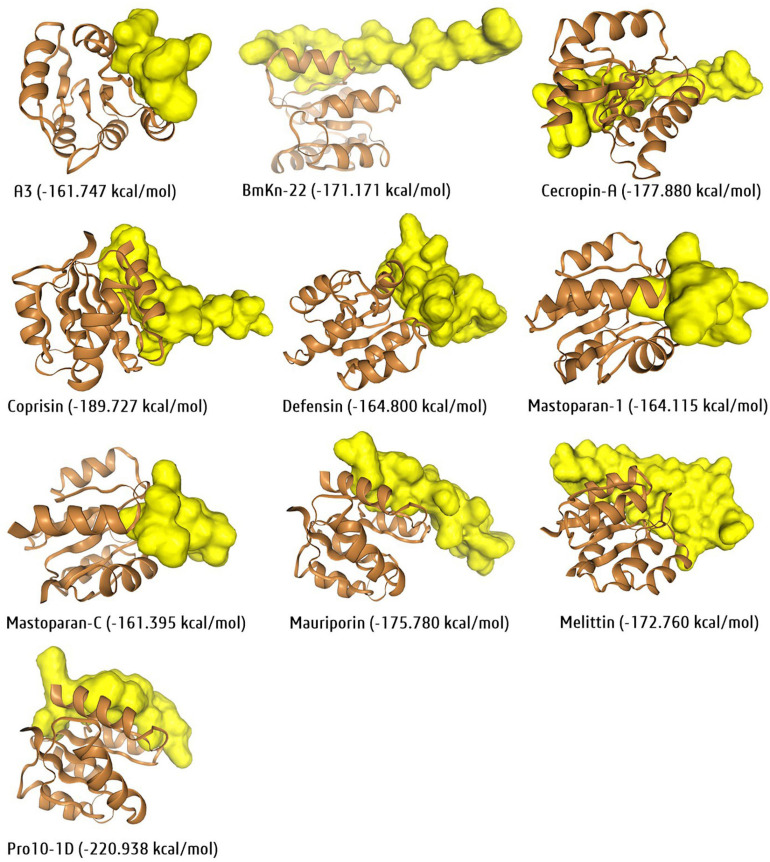
Molecular docking interaction of ten exclusive antibiofilm insect-peptides against the putative biofilm target DNA-binding response regulator/transcriptional factor, LuxR (PDB ID:3B2N) of *S. aureus* by the peptide docking server, HPEPDOCK. Docking score presented in parentheses with a negative sign and more in negative docking score more in potency according to docking tool.

Molecular docking is a technique used to estimate the binding affinity between a target and a ligand, which means any proposed therapeutic agents biding affinity/interaction with a desired target protein of interest. In peptide-based antibiofilm research, molecular docking can predict the binding affinity of any desired peptide against any putative biofilm-associated target enzyme. As we know, bacterial Lux operon encoded genetic factors, LuxR and LuxL are two well-known QS-regulator genes during biofilm formation; thus, as an example, the selective antibiofilm peptides were docked against the protein structure of *S. aureus*-LuxR (PDB ID: 3B2N) and recorded the binding affinity using HPEPDOCK tool with proper interaction (Figure 8). Among all, Pro-10-1D was the most effective peptide against *S. aureus*-LuxR based on the recorded docking score of -220.938 kcal/mol (some docking tool/software represent the docking score in negative sign and some in positive sign based on their algorithms). Similarly, several protein-peptide docking tools, namely, HDOCK, ClusPro PeptiDock, MDockPeP^39^ are also available for molecular docking analysis. Overall, molecular docking is the most widely used tool for potential peptides, drug-able agents and repurposing therapeutic lead section.

**FIGURE 9:**
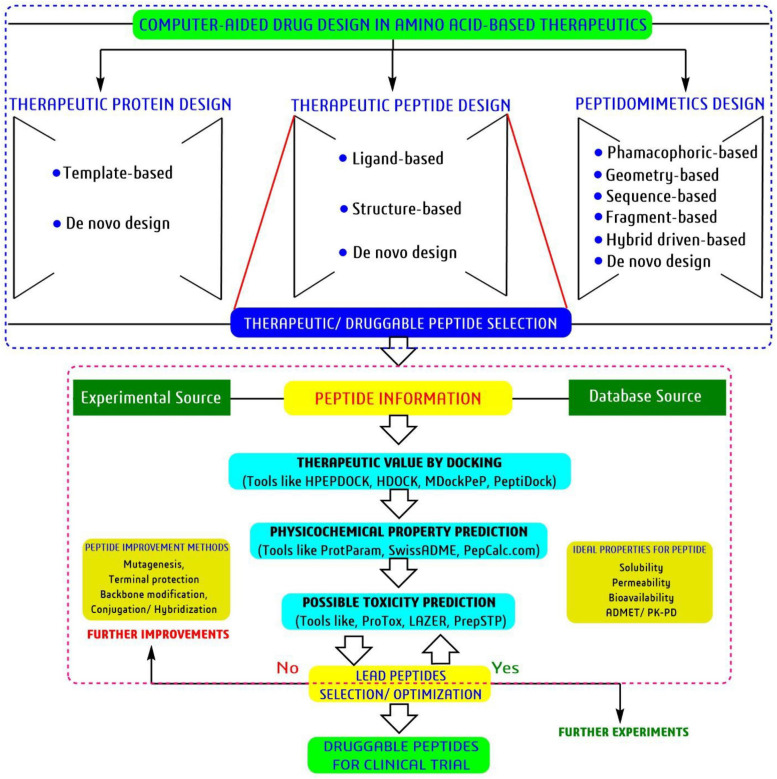
Schematic diagram toward selection and validation of therapeutic peptides using tools of bioinformatics.

Currently, bioinformatics tools can mimicry the whole natural biological system through advanced computational coding and artificial intelligence techniques. Different types of bioinformatics tools are available for individual parameter analysis, like peptide identification to peptide validation to clinical trial information (Lee et al., 2019; Lin et al., 2020). Thus, molecular docking, pharmacophore modeling, and molecular dynamic simulation are the most widely used bioinformatics tools to minimize resource, time, and cost while selecting potential candidates by different pharmaceutical industries and drug developers (Figure 9). Simultaneously, the prediction of such possible biological, physiological, toxicity profiles directly reduce the most expensive experiments and removes the unwanted/unfavorable candidates at the early stage of drug discovery or computer-aided drug discovery (CADD) or machine-learning approach platform (Lin et al., 2020; Mulligan, 2020). However, for every platform and approach, there is a limitation. The CADD platform also contains some demerits. Most tools and software are developed in different algorithms and hypotheses; thus, the same analysis can vary in decision-making. Particularly, there are several challenges such as, solubility, permeability, delivery associated with success of peptide-based drug discovery (Farhadi and Hashemian, 2018; Wagner et al., 2018; Lee et al., 2019). Computational programming can be used to some extent and always not a hundred percent similar to natural biological processes. Thus, bioinformatics tools are most suitable as a guidance/reference for selecting and validating drug candidates, but do not recommend any drug candidates for human consumption. In conclusion, bioinformatics could be a cost-effective essential tool in 21st century for any research analysis and mainly for drug discovery.

## Future Perspective and Concluding Remarks

It is now well evident that the main immune effector molecules of Insects are AMPs. Diversity of insects are huge and there is growing evidence that the natural system of insects is fairly dynamic. At the same time, due to the conserved biological evolution, bioactive molecules and signaling pathways within the natural system of insects exhibit much more similarity with vertebrates (including humans). Researchers worldwide are in search of novel bioactive molecule(s) with novel mechanism of action as antimicrobials. Nevertheless, it is convincing that AMPs could be an innovative antimicrobial candidate. Several factors such as sequence, the charge, the helicity, the amphipathicity and the overall hydrophobicity of AMPs are crucial in considering them as effective antimicrobial agents. However, a few studies have been carried out on insect derived peptides as antimicrobial agents that can prevent formation of biofilm/eradicate the biofilm. It is also obvious that no AMP from insects have been progressed to the pre- clinical and clinical stages of development as therapeutics.

It is evident that peptides show potential antimicrobial activity with several non-specific inhibition mechanisms (Dosler and Karaaslan, 2014; Wu et al., 2018; Raheem and Straus, 2019). Insect-derived antibacterial agents show potential activities against planktonic as well as some biofilm producing pathogenic specific bacterial stains such as uropathogenic *E. coli*, methicillin-resistant *S. aureus*, vancomycin-resistant *S. aureus*, carbapenem-resistant *A. baumannii* and others (Dosler and Karaaslan, 2014; Wu et al., 2018). The present review includes 103 peptides from insect sources with antimicrobial property in planktonic cells but, of these, only seven isolated and three derived peptides were documented against biofilm. However, screening all these AMPs in biofilm models are yet to be investigated. Thus, this review encourages researchers to screen insect derived peptides on different biofilm models. Moreover, more research should be directed toward finding novel antimicrobial peptides from insects.

## Author Contributions

AB, AS, SS, and SP designed the hypothesis. AS and SS performed the computational work. SS analyzed the computationally generated data. SP prepared the draft manuscript. GS and PM commented on the final manuscript. All authors contributed to manuscript revision, read, and approved the submitted version.

## Conflict of Interest

The authors declare that the research was conducted in the absence of any commercial or financial relationships that could be construed as a potential conflict of interest.

## Footnotes

1 https://www.ncbi.nlm.nih.gov/

2 https://www.uniprot.org/

3 https://www.rcsb.org/

4 http://dramp.cpu-bioinfor.org/

5 http://biotechlab.fudan.edu.cn/database/lamp./

6 http://yadamp.unisa.it/about.aspx

7 https://dbaasp.org/home

8 http://www.camp.bicnirrh.res.in/

9 http://www.bicnirrh.res.in/classamp/

10 http://isyslab.info/StraPep/

11 https://zhanglab.ccmb.med.umich.edu/BioLiP/

12 http://www.sbg.bio.ic.ac.uk/∼phyre2/

13 http://bioinf.cs.ucl.ac.uk/psipred/

14 https://zhanglab.ccmb.med.umich.edu/I-TASSER/

15 https://blast.ncbi.nlm.nih.gov/Blast.cgi

16 https://web.expasy.org/protparam/

17 http://www.swissadme.ch/index.php

18 http://cabgrid.res.in:8080/amppred/index.html

19 http://www.baamps.it/

20 http://www.campsign.bicnirrh.res.in/

21 https://bioserv.rpbs.univ-paris-diderot.fr/services/PEP-FOLD3/

22 http://huanglab.phys.hust.edu.cn/hpepdock/

23 http://hdock.phys.hust.edu.cn/

24 https://cluspro.org/home.php

25 https://cluspro.org/peptide/index.php

26 https://pepcalc.com/

27 http://www.tulane.edu/∼biochem/WW/PepDraw/

28 http://biotools.nubic.northwestern.edu/proteincalc.html

29 http://biotriangle.scbdd.com/protein/index/

30 https://www.thermofisher.com/in/en/home/life-science/protein-biology/peptides-proteins/custom-peptide-synthesis-services/peptide-analyzing-tool.html

31 https://www.bioinfor.com/peptide-mutations-homology-searching/

32 https://toolkit.tuebingen.mpg.de/tools/hhpred

33 https://www.ebi.ac.uk/Tools/msa/clustalo/

34 https://www.megasoftware.net/

35 https://swissmodel.expasy.org/

36 http://www.cbs.dtu.dk/services/CPHmodels/

37 https://bioserv.rpbs.univ-paris-diderot.fr/services/PEP-FOLD3/

38 https://zougrouptoolkit.missouri.edu/mdockpep/index.html

## Abbreviations

ADMET: absorption, distribution, metabolism, excretion, and toxicity
AMPs: antimicrobial peptides
Bap: biofilm associated protein
BP: bacterial prostatitis
BPs: bioactive peptides
BV: bacterial vaginosis
CecA: cecropin A
c-di-GMP: bis-(3′-5′)-cyclic di-guanosine monophosphate
CF: cystic fibrosis
COPD: chronic obstructive pulmonary disease
cylA: cytolysin A
ebp: endocarditis biofilm-associated protein
EPS: exopolysaccharide
ESKAPE: *Enterococcus faecalis, Staphylococcus aureus, Klebsiella pneumoniae, Acinetobacter baumannii, Pseudomonas aeruginosa* and *Enterobacter*
Esp: enterococcal surface protein
fib: fibrinogen−binding protein
FISH: fluorescence microscopy and fluorescence in situ hybridization
GP: Gram-positive
IBD: crohn’s disease
MIC: minimum inhibitory concentration
MRSA: methicillin-resistant *S. aureus;*
NAL: nalidixic acid
NCBI: National Center for Biotechnology Information
OM: otitis media
PDB: protein data bank
PIA: intercellular adhesin
PNAG: polymeric N-acetyl-glucosamine
QS: quorum sensing
sprE: serine protease
sRNAs: small RNAs
UPEC: uropathogenic E. *coli*
UTI: urinary tract infection.
