# Supplementary material for: Antimicrobial Peptides Derived From Insects Offer a Novel Therapeutic Option to Combat Biofilm: A Review
Source: Front Microbiol. 2021 Jun 10;12:661195. doi: 10.3389/fmicb.2021.661195 (PMC8265172; doi:10.3389/fmicb.2021.661195)
Supplement: Supplementary file 1 [file Data_Sheet_1.docx]

**Antimicrobial peptides derived from insects offer a novel therapeutic option to combat biofilm: A systematic review**

**Alaka Sahoo ^a^, Shasank Sekhar Swain ^b^, Ayusman Behera ^c^, Gunanidhi Sahoo^d^, Pravati Kumari Mahapatra^d^ and Sujogya Kumar Panda^e*^**

^a^ Department of Skin & VD, Institute of Medical Sciences & SUM Hospital, Siksha ‘O’ Anusandhan Deemed to be University, Bhubaneswar-751003, Odisha, India

^b^ Division of Microbiology & NCDs, ICMR-Regional Medical Research Centre, Bhubaneswar-751023, Odisha, India

^c^ Department of Zoology, Maharaja Sriram Chandra Bhanja Deo University, Takatpur, Baripada-757003, Odisha, India

^d^ Department of Zoology, Utkal University, Vani Vihar, Bhubaneswar-751004, Odisha, India

^e^ Centre of Environment, Climate Change and Public Health, RUSA 2.0, Utkal University, Vani Vihar, Bhubaneswar-751004, Odisha, India

* Correspondence: SKP, email-sujogyapanda@gmail.com

Supplementary Material

**Table S1**. List of antibacterial peptides isolated from different insects.

| Sl. No. | Peptide | Source | Anti-biofilm | MIC | Reference |
| --- | --- | --- | --- | --- | --- |
|  | AamAP1 | *Androctonus amoeruxi* | *Staphylococcus aureus* and *Escherichia coli* | 20 and 150 µM | (Almaaytah et al., 2012) |
|  | AamAP2 | *Androctonus amoeruxi* | *S. aureus* and *E. coli* | 20 and 150 µM | (Almaaytah et al., 2012) |
|  | Abaecin | *Bombus pascuorum* | *E. coli* | 20 µM | (Rahnamaeian et al., 2016) |
|  | Acaloleptin A1 | *Acalolepta luxuriosa* | *E. coli* | 12.76 µg/ml | (Imamura et al., 1999) |
|  | Acaloleptin A2 | *Acalolepta luxuriosa* | *E. coli* | 13.76 µg/ml | (Imamura et al., 1999) |
|  | Acaloleptin A3 | *Acalolepta luxuriosa* | *E. coli* | 11.76 µg/ml | (Imamura et al., 1999) |
|  | AaeAP1 | Androctonus aeneas | *S. aureus* and *E. coli* | 16 and >512 mg/L | (Du et al., 2015) |
|  | AaeAP2 | Androctonus aeneas | *S. aureus* | 32 mg/L | (Du et al., 2015) |
|  | Apidaecin Ia | *Apis mellifera* | *E. coli,*  *Klebsiella pneumoniae,*  *Pseudomonas aeruginosa,*  *Salmonella enteritidis,*  *Salmonella typhimurium* | 0.02-0.2 µg/ml | (Casteels et al., 1989) |
|  | Apidaecin Ib | *Apis mellifera* | *E. coli, K. pneumoniae, P. aeruginosa, S. enteritidis, S. typhimurium* | 0.02-0.2 µg/ml | (Casteels et al., 1989) |
|  | Apidaecin II | *Apis mellifera* | *E. coli, K. pneumoniae, P. aeruginosa, S. enteritidis, S. typhimurium* | 0.02-0.5 µg/ml | (Casteels et al., 1989) |
|  | Attacin | *Hyalophora cecropia* | *E. coli* | 0.5-1 µM | (Hultmark et al., 1983) |
|  | Attacin A | *Glossina morsitans* | *E. coli, Enterobacter cloacae, K. pneumoniae* | 24-72.7 μg/ml | (Xie et al., 2009) |
|  | Attacin AP1 | *Glossina morsitans* | *E. coli, E. cloacae, K. pneumoniae* | 4.2-51.6 μg/ml | (Xie et al., 2009) |
|  | Attacin B | *Hyalophora cecropia* | *E. coli, Pseudomonas maltophilia,* *Acinetobacter calcoaceticus,* *E. cloacae, Bacillus megaterium* | 0.6-15 µM | (Hultmark et al., 1983) |
|  | Attacin C | *Drosophila melanogaster* | *E. coli, S. typhimurium, P. aeruginosa, S. aureus,* | > 200 µM | (Rabel et al., 2004) |
|  | Attacin E | *Hyalophora cecropia* | *E. coli, P. maltophilia, A. calcoaceticus, E. cloacae, B. megaterium* | 1-20 µM | (Hultmark et al., 1983) |
|  | BmKn 2 | *Mesobuthus martensii* | *S. aureus, Bacillus subtilis, P. aeruginosa, Bacillus thuringiensis* | 6.25-50 μg/ml | (Cao et al., 2012a) |
|  | Cobatoxin | Galleria mellonella | Micrococcus luteus | 120 μm | (Bolouri Moghaddam et al., 2016) |
|  | Coprisin | *Copris tripartitus* | *E. coli, S. typhimurium, P. aeruginosa, S. aureus, B. subtilis, S. epidermidis* | 0.50-16 μM | (Lee et al., 2013) |
|  | Cecropin | *Musca domestica* | *E. coli* | 1.56 µM | (Lehrer and Lu, 2012) |
|  | Cecropin A | Galleria mellonella | *Acinetobacter baumannii, E. coli, M. luteus* | 0.5-32 µM | (Giacometti et al., 2003) |
|  | Cecropin A2 | *Aedes aegypti* | *P. aeruginosa* and other Gram-negative bacteria | 2 to 64 μg/ml | (Zheng et al., 2017) |
|  | Cecropin B | *Hyalophora cecropia* | *E. coli, Bacillus fragilis, P. aeruginosa, S. epidermidis* | 1.7-33.3 µM | (Moore et al., 1996) |
|  | Cecropin B_1_ | *Bombyx mori* | *Acinetobacter* sp*., E. coli, Pseudomonas fluorescens, S. aureus, S. pyogenes, S, epidermidis, B. subtilis, P. aeruginosa* | 0.49-16 µM | (Hara and Yamakawa, 1995a) |
|  | Cecropin B2 | *Bombyx mori* | *E. coli, B. megaterium, P. aeruginosa, S. marcescens* | 0.35-17.2 µM | (Gazit et al., 1994) |
|  | Cecropin P_1_ | *Hyalophora cecropia* | *E. coli, P. aeruginosa* | 3.3 and 8.3 µM | (Moore et al., 1996) |
|  | Cecropin D | *Bombyx mori* | *E. coli* | 6.25 µg/ml | (Hara and Yamakawa, 1995b) |
|  | Cecropin-like peptide 1/ CLP 1 | *Hermetia illucens* | *E. coli, E. aerogenes, P. aeruginosa* | 0.52-2.07 µg/ml | (Park and Yoe, 2017) |
|  | Coleoptericin A | *Allomyrina dichotoma* | *S. aureus, E. coli, B. subtilis* | 3.1-31.9 µg/ml (LC_50_) | (Sagisaka et al., 2001) |
|  | Coleoptericin B | *Allomyrina dichotoma* | *S. aureus, E. coli, B. subtilis* | 2.8-32 LC50 µg/ml | (Sagisaka et al., 2001) |
|  | Coleoptericin C (insect defensin) | *Zophob utrutus* | *M. luteus and E. coli, Corynebacterium* sp. | 2-10 µg/ml | (Bulet et al., 1991) |
|  | Defensin (51aa) | *Anopheles gambiae* | *Aerococcus viridans, B. cereus, B. subtilis, E. faecalis, Listeria monocytogenes, S. aureus, E. coli, Streptococcus  pyogenes* | 0.012-25 µM | (Vizioli et al., 2001) |
|  | Defensin-like peptide/ DLP4 | *Hermetia illucens* | *S. aureus*, *S. epidermidis, E. coli, B. subtilis* | 0.02-1.17 μM | (Park et al., 2015) |
|  | Diptericin A | *Phormia terranovae* | *B. subtilis, E. coli,*  *B. thuringiensis,*  *E. cloacae, P. aeruginosa, Micrococcus luteus* | 0.75-3 µg/ml | (Keppi et al., 1989)  ( |
|  | Drosocin | *Drosophila melanogaster* | *E. coli, M. luteus* | 75 and 500 nM | (Bulets et al., 1993) |
|  | Gallerimycin | Galleria mellonella | *M. luteus* | 60 µM | (Bolouri Moghaddam et al., 2016) |
|  | Gloverin | *Hyalophora phora* | *E. coli* | 1-3 µM | (Axen et al., 1997) |
|  | Gloverin  glycine-rich peptide | *Antheraea assamensis* | *E. coli* and *E. cloacae* | 16.24 & 18.56 µg/ml | (Nayak et al., 2018) |
|  | Gm-prolinerich peptide 1 | *Galleria mellonella* | *M. luteus* | 31.4-55 µM | (Cytryńska et al., 2007) |
|  | Gm-defensinlike peptide | *Galleria mellonella* | *S. lutea* | 1.4-1.9 µM | (Cytryńska et al., 2007) |
|  | Gm-anionic peptide 1 | *Galleria mellonella* | *M. luteus, L. monocytogenes* | 11.4-22.7, 45.5-90.9 µM | (Cytryńska et al., 2007) |
|  | Gm-anionic peptide 2 | *Galleria mellonella* | *M. luteus* | 43.3-86.6 µM | (Cytryńska et al., 2007) |
|  | Gm-cecropin D-like peptide | *Galleria mellonella* | *E. coli* | 6.9-8.6 µM | (Cytryńska et al., 2007) |
|  | Heterin 1 | *Heterometrus spinifer* | *B. megaterium, M. luteus, and S. aureus, E. coli, P. fluorences, P. putida* | 4-42 µM | (Wu et al., 2014) |
|  | Heterin 2 | *Heterometrus spinifer* | *B. megaterium, M. luteus, S. aureus, E. coli, P. fluorences, P. putida, K. oxytoca, S. enterica* | 5.6- > 45 µM | (Wu et al., 2014) |
|  | Hinnavin I | *Artogeia rapae* | *E. coli, E. cloacae, B. megaterium, B. thuringiensis, M. luteus* | 0.3-0.5 µg/ml | (Bang and Yoe, 1997) |
|  | Hinnavin II | *Artogeia rapae* | *E. coli, E. cloacae, B. megaterium, B. thuringiensis, M. luteus* | 25 µM | (Yoe et al., 2006) |
|  | Holotricin 1 | *Holotrichia diomphalia* | *S. aureus, E. coli, B. subtilis, M. luteus, S. pyrogen, S. enterica* | 0.3-4.3 µg/ml | (Lee and Lee, 1996) |
|  | Holotricin 1A | *Holotrichia diomphalia* | *S. aureus, E. coli, B. subtilis, M. luteus, S. pyrogen* | 0.5-4 µg/ml | (Lee and Lee, 1996) |
|  | Holotricin 1B | *Holotrichia diomphalia* | *S. aureus, E. coli, B. subtilis, M. luteus, S. pyrogen* | 0.5-4 µg/ml | (Lee and Lee, 1996) |
|  | Holotricin 1C | *Holotrichia diomphalia* | *S. aureus, E. coli, B. subtilis, M. luteus, S. pyrogen* | 5->30 µg/ml | (Lee and Lee, 1996) |
|  | HsAp | *Heterometrus spinifer* | *S. enterica, E. coli, B. megaterium, B. thuringiensis* | 11.8–51.2 µM | (Nie et al., 2012) |
|  | Jelleine-I | *Heterometrus spinifer* | *S. aureus, S. saprophyticus, B. subtilis, B. cereus, E. coli, K. pneumoniae, P. aeruginosa* | 2.5-15 µg/ml | (Fontana et al., 2004) |
|  | Jelleine-II | *Heterometrus spinifer* | *S. aureus, S. saprophyticus, B. subtilis, B. cereus, E. coli, K. pneumoniae, P. aeruginosa* | 10-30 µg/ml | (Fontana et al., 2004) |
|  | Jelleine-III | *Heterometrus spinifer* | *S. aureus, S. saprophyticus, B. subtilis, B. cereus, E. coli, K. pneumoniae, P. aeruginosa* | 15-30 µg/ml | (Fontana et al., 2004) |
|  | Lebocin | *Bombyx mori* | *E. coli* | 10 ug/ml | (Hara and Yamakawa, 1995a) |
|  | Lebocin 3 | *Bombyx mori* | *E. coli* | 800 µg/ml | (Hara and Yamakawa, 1995a) |
|  | Lucifensin (40aa) | *Lucilia sericata* | *E. coli, S. aureus* (MRSA)*, S. pyogenes, S. pneumoniae, P. aeruginosa* | 2 - >128 mg | (Andersen et al., 2010) |
|  | Lucilin | *Lucilia sericata* | *E. coli, E. cloacae, S. typhi* | 18-50 µg/ml | (Téllez and Castaño-Osorio, 2014) |
|  | Mauriporin | *Androctonus mauritanicus* | *P. aeruginosa, S. epidermidis*  *L. ivanovii, E. coli, S. enterica, S. typhimurium, P. aeruginosa, A. baumannii, K. pneumoniae* | 5-10 µM | (Almaaytah et al., 2014) |
|  | Melittin | *Apis mellifera*  *Apis cerana* | *A. baumannii, P. aeruginosa,*  *Staphylococcus haemolyticus, K. pneumoniae* | 0.5-32 µg/ml | (Giacometti et al., 2003) |
|  | Metchnikowin | *Drosophila melanoguster* | *M. luteus* | 0.5-1 µM | (Levashina et al., 1995) |
|  | Moricin | *Bombyx mori* | *Acinetobacter* spp., *E. coli, P. fluorescens, S. aureus, S. pyogenes, S. epidermidis, B. subtilis, P. aeruginosa* | 0.18-0.81 µM | (Hara and Yamakawa, 1995b) |
|  | Opisin | *Opistophthalmus glabrifrons* | *S. aureus,* MRSA, *B.* *megaterium, B. thuringiensis, N. coralline, M. luteus* | 4-10 µM | (Bao et al., 2015) |
|  | Oxysterlin 1 | *Oxysternon conspicillatum* | *E. coli, K. pneumoniae, S. typhimurium, E. cloacae, P. aeruginosa* | 6.25-50 µg/ml | (Segovia et al., 2017) |
|  | Oxysterlin 2 | *Oxysternon conspicillatum* | *E. coli, K. pneumoniae* | 25-50 µg/ml | (Segovia et al., 2017) |
|  | Oxysterlin 3 | *Oxysternon conspicillatum* | *E. coli, K. pneumoniae, S. typhimurium, E. cloacae, P. aeruginosa* | 3.12-12.5 µg/ml | (Segovia et al., 2017) |
|  | Oxysterlin 4 | *Oxysternon conspicillatum epidermides* | *E. coli, K. pneumoniae, S. typhimurium, E. cloacae, P. aeruginosa* | >200 μg/mL | (Segovia et al., 2017) |
|  | Pandinin 2 | *Pandinus imperator* | *E. coli* and *S. aureus* | 18.8 and 37.5 µM | (Rodríguez et al., 2014) |
|  | Pantinin 1 | *Pandinus imperator* | *S. aureus,* MRSA, VRE, *B. megaterium, M. luteus* | 8-32 µM | (Wu et al., 2014) |
|  | Pantinin 2 | *Pandinus imperator* | *S. aureus,* MRSA, VRE, *B. megaterium, M. luteus* | 28-48 µM | (Wu et al., 2014) |
|  | Pantinin 3 | *Pandinus imperator* | *S. aureus,* MRSA, VRE, *B. megaterium, M. luteus* | 4-16 µM | (Zeng et al., 2013) |
|  | Papiliocin (38aa) | *Papilio xuthus* | E. coli, S. epidermidis, *B. subtills*, *P. aeruginosa,* | 0.5-32 µM | (Kim et al., 2011) |
|  | Persulcatusin | *Ixodes persulcatus* | *S. aureus* (MRSA), *S. aureus* (VRSA) | 1-8 μg/mL | (Miyoshi et al., 2017)  (Miyoshi et al., 2016) |
|  | Ponericins | *Pachycondyla goeldii* | E. coli, S. aureus, P. aeruginosa, B. stearothermophilus, K. pneumoniae | 8-30 μg/mL | (Orivel et al., 2001) |
|  | Prolixicin | *Rhodnius prolixus* | E. coli, S. *epidermidis*, P. *fluorescens*, B. *subtilis*, E. *aerogenes* | 1.6- > 25 µM | (Ursic-Bedoya et al., 2011) |
|  | Protaetin 2 | *Protaetia brevitarsis* | *M. luteus, B. subtills, B. thuringiensis, E. coli, S. typhimurium, S. marcescens* | 0.29-33.24 µg/ml | (Yoon, et al., 2003) |
|  | Pyrrhocoricin | *Pyrrhocoris apterus* | *M. luteus, B. megaterium, S. aureus, E. coli, P. aeruginosa, K. pneumoniae* | 1- >10 µM | (Cociancich et al., 1994) |
|  | Royalisin | *Apis mellifera* | *P. aeruginosa, S. aureus, S. alactolyticus, S. inttermedius,* *B, S. xylosus, S. cholearasuis* | 4-11 µg/ml | (Bílikova et al., 2015) |
|  | Sapecin | *Sarcophaga peregrine* | *E. coli, P. mirabilis, K. pneumoniae, S. aureus, S. mutans, B. megaterium* | 0.2 - > 20 µg/ml | (Yamada and Natori, 1993) |
|  | Sapecin B | *Sarcophaga peregrine* | *E. coli, P. mirabilis, K. pneumoniae, S. aureus, Streptococcus  mutans, B. megaterium* | 0.2 - > 20 µg/ml | (Yamada and Natori, 1993) |
|  | Sapecin C | *Sarcophaga peregrine* | *E. coli, P. mirabilis, K. pneumoniae, S. aureus, S. mutans, B. megaterium* | 0.2 - > 20 µg/ml | (Yamada and Natori, 1993) |
|  | Sarconesin II | *Sarconesiopsis magellanica* | *E. coli, P. aeruginosa,* *S. aureus, M. luteus* | 1.9-15.6 µM | (Díaz-Roa et al., 2019) |
|  | Sarcotoxin Pd | *Paederus dermatitis* | *E. coli, K. pneumoniae, E. cloacae, B. subtilis P. aeruginosa,* *S. aureus* | 6.12-19.24 µg/ml | (Saito et al., 2005) |
|  | Sarcotoxins IA | *Sarcophaga peregrine* | *E. coli, K. pneumoniae, Shigella sonnei, P, vulgaris* | 0.15-0.3 µg/mL | (Okada and Natori, 1985) |
|  | Sarcotoxins IB | *Sarcophaga peregrine* | *E. coli, K. pneumoniae, S. sonnei, P, vulgaris* | 0.15-0.3 µg/mL | (Okada and Natori, 1985) |
|  | Sarcotoxins IC | *Sarcophaga peregrine* | *E. coli, K. pneumoniae, S. sonnei, P, vulgaris* | 0.15-0.3 µg/mL | (Okada and Natori, 1985) |
|  | Smp 24 | *Maurus palmatus* | *B. subtilis, S. epidermidis, S, aureus, E. coli, P. aeruginosa* | 4-128 µg/ml | (Harrison et al., 2016) |
|  | Smp 43 | *Maurus palmatus* | *B. subtilis, S. epidermidis, S, aureus, E. coli, P. aeruginosa* | 8-128 µg/ml | (Abdel-Rahman et al., 2013) |
|  | Spiniferin | *Heterometrus spinifer* | *S. aureus, E. coli, B. subtilis, M. luteus, S. pyrogen, S. enterica* | 42- >82 µM | (Wu et al., 2014) |
|  | Spodopsin Ia (35aa) | *Spodoptera litura* | *E. coli* Ek132  *E. coli* K12 | 0.1-0.38 mg/ml | (Yi et al., 2014) |
|  | Spodopsin Ib (35aa) | *Spodoptera litura* | *E. coli* Ek132  *E. coli* K12 | 0.1-0.38 mg/ml | (Yi et al., 2014) |
|  | StCT2 | *Scorpiops tibetanus* | *B. thuringiensis, S. aureus, B. subtilis, M. luteus, S. epidermidis* | 6.25–25 lg/mL | (Cao et al., 2012b) |
|  | Stigmurin | *Tityus stigmurus* | S. aureus and MRSA | 8.7 to 69.5 mM | (De Melo et al., 2015) |
|  | Stomoxyn (41aa) | *Stomoxys calcitrans* | *M. luteus, E. coli* and several other bacteria | 5-10 µg/L | (Landon et al., 2006) |
|  | Tenecin 1 (43aa) | *Tenebrio molitor* | *E. coli, B. subtilis, S. aureus, S. pyrogen, M. luteus* | 0.8-8 µg/ml | (Moon et al., 1994) |
|  | Tenecin 4 | *Tenebrio molitor* | *E. coli* | 0.5 µg/ml | (Chae et al., 2012) |
|  | TsAP 1 | *Tityus serrulatus* | *S. aureus, E. coli* | 120-160 µM | (Guo et al., 2013) |
|  | TsAP 2 | *Tityus serrulatus* | *S. aureus* | 5 µM | (Guo et al., 2013) |
|  | Vejovine | *Vaejovis mexicanus* | *A. baumannii, E. coli,* *K. pneumoniae, P. aeruginosa* | 5-50 µM | (Hernández-Aponte et al., 2011) |
|  | VmCT1 | *Vaejovis mexicanus smithi* | *S. aureus, B. subtilis, E. coli, S. typhi, P. aeruginosa, S. agalactiae* | 5-25 µM | (Ramírez-Carreto et al., 2015) |
|  | VmCT2 | *Vaejovis mexicanus smithi* | *S. aureus, B. subtilis, E. coli, S. typhi, P. aeruginosa, S. agalactiae* | 5-25 µM | (Ramírez-Carreto et al., 2015) |

**References**

Abdel-Rahman, M. A., Quintero-Hernandez, V., and Possani, L. D. (2013). Venom proteomic and venomous glands transcriptomic analysis of the Egyptian scorpion Scorpio maurus palmatus (Arachnida: Scorpionidae). *Toxicon* 74, 193–207. doi:10.1016/j.toxicon.2013.08.064.

Almaaytah, A., Tarazi, S., Alsheyab, F., Al-Balas, Q., and Mukattash, T. (2014). Antimicrobial and antibiofilm activity of mauriporin, a multifunctional scorpion venom peptide. *Int. J. Pept. Res. Ther.* 20, 397–408. doi:10.1007/s10989-014-9405-0.

Almaaytah, A., Zhou, M., Wang, L., Chen, T., Walker, B., and Shaw, C. (2012). Antimicrobial/cytolytic peptides from the venom of the North African scorpion, Androctonus amoreuxi: Biochemical and functional characterization of natural peptides and a single site-substituted analog. *Peptides* 35, 291–299. doi:10.1016/j.peptides.2012.03.016.

Andersen, A. S., Sandvang, D., Schnorr, K. M., Kruse, T., Neve, S., Joergensen, B., et al. (2010). A novel approach to the antimicrobial activity of maggot debridement therapy. *J. Antimicrob. Chemother.* 65, 1646–1654. doi:10.1093/jac/dkq165.

Axen, A., Carlsson, A., Engstrom, A., and Bennich, H. (1997). Gloverin, an Antibacterial Protein from the Immune Hemolymph of Hyalophora Pupae. *Eur. J. Biochem.* 247, 614–619. doi:10.1111/j.1432-1033.1997.00614.x.

Bang, I. S., and Yoe, S. Y. S. and S. M. (1997). Hinnavin I, an Antibacterial Peptide from Cabbage Butterfly, Artogeia rapae. *Mol. Cells* 7, 509–513. Available at: http://www.molcells.org/journal/view.html?spage=509&volume=7&number=4 [Accessed January 9, 2021].

Bao, A., Zhong, J., Zeng, X.-C., Nie, Y., Zhang, L., and Peng, Z. F. (2015). A novel cysteine-free venom peptide with strong antimicrobial activity against antibiotics-resistant pathogens from the scorpion *Opistophthalmus glabrifrons*. *J. Pept. Sci.* 21, 758–764. doi:10.1002/psc.2801.

Bílikova, K., Huang, S. C., Lin, I. P., Šimuth, J., and Peng, C. C. (2015). Structure and antimicrobial activity relationship of royalisin, an antimicrobial peptide from royal jelly of Apis mellifera. *Peptides* 68, 190–196. doi:10.1016/j.peptides.2015.03.001.

Bolouri Moghaddam, M. R., Tonk, M., Schreiber, C., Salzig, D., Czermak, P., Vilcinskas, A., et al. (2016). The potential of the Galleria mellonella innate immune system is maximized by the co-presentation of diverse antimicrobial peptides. *Biol. Chem.* 397, 939–945. doi:10.1515/hsz-2016-0157.

Bulet, P., Cociancich, S., Dimarcq, J.-L., Lambert, J., Reichhart, J.-M., Hoffmann, D., et al. (1991). Insect immunity. Isolation from a coleopteran insect of a novel inducible antibacterial peptide and of new members of the insect defensin family. *J. Biol. Chem.* 266, 24520–24525.

Bulets, P., Dimarcqs, J.-L., Hetru, C., Lagueux, M., Charlet, M., Hegy5 Ll, G., et al. (1993). THE JOURNAL OF BIOLOGICAL CHEMISTRY A Novel Inducible Antibacterial Peptide of Drosophila Carries an 0-Glycosylated Substitution*. *J. Biol. Chem.* 268, 14893–14697.

Cao, L., Dai, C., Li, Z., Fan, Z., Song, Y., Wu, Y., et al. (2012a). Antibacterial Activity and Mechanism of a Scorpion Venom Peptide Derivative In Vitro and In Vivo. *PLoS One* 7, e40135. doi:10.1371/journal.pone.0040135.

Cao, L., Li, Z., Zhang, R., Wu, Y., Li, W., and Cao, Z. (2012b). StCT2, a new antibacterial peptide characterized from the venom of the scorpion Scorpiops tibetanus. *Peptides* 36, 213–220. doi:10.1016/j.peptides.2012.04.010.

Casteels, P., Ampe, C., Jacobs, F., Vaeck, M., and Tempst, P. (1989). Apidaecins: antibacterial peptides from honeybees. *EMBO J.* 8, 2387–2391. doi:10.1002/j.1460-2075.1989.tb08368.x.

Chae, J. H., Kurokawa, K., So, Y. I., Hwang, H. O., Kim, M. S., Park, J. W., et al. (2012). Purification and characterization of tenecin 4, a new anti-Gram-negative bacterial peptide, from the beetle Tenebrio molitor. *Dev. Comp. Immunol.* 36, 540–546. doi:10.1016/j.dci.2011.09.010.

Cociancich, S., Dupont, A., Hegy, G., Lanot, R., Holder, F., Hetru, C., et al. (1994). Novel inducible antibacterial peptides from a hemipteran insect, the sap-sucking bug Pyrrhocoris apterus. *Biochem. J.* 300, 567–575. doi:10.1042/bj3000567.

Cytryńska, M., Mak, P., Zdybicka-Barabas, A., Suder, P., and Jakubowicz, T. (2007). Purification and characterization of eight peptides from Galleria mellonella immune hemolymph. *Peptides* 28, 533–546. doi:10.1016/j.peptides.2006.11.010.

De Melo, E. T., Estrela, A. B., Santos, E. C. G., Machado, P. R. L., Farias, K. J. S., Torres, T. M., et al. (2015). Structural characterization of a novel peptide with antimicrobial activity from the venom gland of the scorpion Tityus stigmurus: Stigmurin. *Peptides* 68, 3–10. doi:10.1016/j.peptides.2015.03.003.

Díaz-Roa, A., Espinoza-Culupú, A., Torres-García, O., Borges, M. M., Avino, I. N., Alves, F. L., et al. (2019). Sarconesin II, a New Antimicrobial Peptide Isolated from Sarconesiopsis magellanica Excretions and Secretions. *Molecules* 24, 2077. doi:10.3390/molecules24112077.

Du, Q., Hou, X., Wang, L., Zhang, Y., Xi, X., Wang, H., et al. (2015). AaeAP1 and AaeAP2: Novel Antimicrobial Peptides from the Venom of the Scorpion, Androctonus aeneas: Structural Characterisation, Molecular Cloning of Biosynthetic Precursor-Encoding cDNAs and Engineering of Analogues with Enhanced Antimicrobial and Anticancer Activities. *Toxins (Basel).* 7, 219–237. doi:10.3390/toxins7020219.

Fontana, R., Mendes, M. A., De Souza, B. M., Konno, K., César, L. M. M., Malaspina, O., et al. (2004). Jelleines: A family of antimicrobial peptides from the Royal Jelly of honeybees (Apis mellifera). *Peptides* 25, 919–928. doi:10.1016/j.peptides.2004.03.016.

Gazit, E., Shai, Y., Lee, W. J., and Brey, P. T. (1994). Mode of Action of the Antibacterial Cecropin B2: A Spectrofluorometric Study. *Biochemistry* 33, 10681–10692. doi:10.1021/bi00201a016.

Giacometti, A., Cirioni, O., Kamysz, W., D’Amato, G., Silvestri, C., Del Prete, M. S., et al. (2003). Comparative activities of cecropin A, melittin, and cecropin A-melittin peptide CA(1-7)M(2-9)NH2 against multidrug-resistant nosocomial isolates of Acinetobacter baumannii. *Peptides* 24, 1315–1318. doi:10.1016/j.peptides.2003.08.003.

Guo, X., Ma, C., Du, Q., Wei, R., Wang, L., Zhou, M., et al. (2013). Two peptides, TsAP-1 and TsAP-2, from the venom of the Brazilian yellow scorpion, Tityus serrulatus: Evaluation of their antimicrobial and anticancer activities. *Biochimie* 95, 1784–1794. doi:10.1016/j.biochi.2013.06.003.

Hara, S., and Yamakawa, M. (1995a). Cooperative Antibacterial Relationship between Lebocin and Cecropin D, Antibacterial Peptides Isolated from the Silkworm, Bombyx mori (Lepidoptera: Bombycidae). *Appl. Entomol. Zool.* 30, 606–608. doi:10.1303/aez.30.606.

Hara, S., and Yamakawa, M. (1995b). Moricin, a novel type of antibacterial peptide isolated from the silkworm, Bombyx mori. *J. Biol. Chem.* 270, 29923–29927. doi:10.1074/jbc.270.50.29923.

Harrison, P. L., Abdel-Rahman, M. A., Strong, P. N., Tawfik, M. M., and Miller, K. (2016). Characterisation of three alpha-helical antimicrobial peptides from the venom of Scorpio maurus palmatus. *Toxicon* 117, 30–36. doi:10.1016/j.toxicon.2016.03.014.

Hernández-Aponte, C. A., Silva-Sanchez, J., Quintero-Hernández, V., Rodríguez-Romero, A., Balderas, C., Possani, L. D., et al. (2011). Vejovine, a new antibiotic from the scorpion venom of Vaejovis mexicanus. *Toxicon* 57, 84–92. doi:10.1016/j.toxicon.2010.10.008.

Hultmark, D., Engström, A., Andersson, K., Steiner, H., Bennich, H., and Boman, H. G. (1983). Insect immunity. Attacins, a family of antibacterial proteins from Hyalophora cecropia. *EMBO J.* 2, 571–576. doi:10.1002/j.1460-2075.1983.tb01465.x.

Imamura, M., Wada, S., Koizumi, N., Kadotani, T., Yaoi, K., Sato, R., et al. (1999). Acaloleptins A: Inducible antibacterial peptides from larvae of the beetle,Acalolepta luxuriosa. *Arch. Insect Biochem. Physiol.* 40, 88–98. doi:10.1002/(SICI)1520-6327(1999)40:2<88::AID-ARCH3>3.0.CO;2-B.

Keppi, E., Pugsley, A. P., Lambert, J., Wicker, C., Dimarcq, J.-L., Hoffmann, J. A., et al. (1989). Mode of action of diptericin A, a bactericidal peptide induced in the hemolymph ofPhormia terranovae larvae. *Arch. Insect Biochem. Physiol.* 10, 229–239. doi:10.1002/arch.940100306.

Kim, J. K., Lee, E., Shin, S., Jeong, K. W., Lee, J. Y., Bae, S. Y., et al. (2011). Structure and function of papiliocin with antimicrobial and anti-inflammatory activities isolated from the swallowtail butterfly, Papilio xuthus. *J. Biol. Chem.* 286, 41296–41311. doi:10.1074/jbc.M111.269225.

Landon, C., Meudal, H., Boulanger, N., Bulet, P., and Vovelle, F. (2006). Solution structures of stomoxyn and spinigerin, two insect antimicrobial peptides with an α-helical conformation. *Biopolymers* 81, 92–103. doi:10.1002/bip.20370.

Lee, E., Kim, J. K., Shin, S., Jeong, K. W., Shin, A., Lee, J., et al. (2013). Insight into the antimicrobial activities of coprisin isolated from the dung beetle, Copris tripartitus, revealed by structure-activity relationships. *Biochim. Biophys. Acta - Biomembr.* 1828, 271–283. doi:10.1016/j.bbamem.2012.10.028.

Lee, S. Y., and Lee, Y. U. L. and B. L. (1996). Purification and Characterization of a Holotricin 1 Homologues from Holotrichia diomphalia Larvae. *Mol. Cells* 6, 86–90. Available at: http://www.molcells.org/journal/view.html?spage=86&volume=6&number=1 [Accessed December 30, 2020].

Lehrer, R. I., and Lu, W. (2012). α-Defensins in human innate immunity. *Immunol. Rev.* 245, 84–112. doi:10.1111/j.1600-065X.2011.01082.x.

Levashina, E. A., Ohresser, S., Bulet, P., Reichhart, J. ‐M, Hetru, C., and Hoffmann, J. A. (1995). Metchnikowin, a Novel Immune‐Inducible Proline‐Rich Peptide from Drosophila with Antibacterial and Antifungal Properties. *Eur. J. Biochem.* 233, 694–700. doi:10.1111/j.1432-1033.1995.694_2.x.

Miyoshi, N., Isogai, E., Hiramatsu, K., and Sasaki, T. (2017). Activity of tick antimicrobial peptide from Ixodes persulcatus (persulcatusin) against cell membranes of drug-resistant Staphylococcus aureus. *J. Antibiot. (Tokyo).* 70, 142–146. doi:10.1038/ja.2016.101.

Moon, H. J., Lee, S. Y., Kurata, S., Natori, S., and Lee, B. L. (1994). Purification and molecular cloning of cDNA for an inducible antibacterial protein from larvae of the coleopteran, Tenebrio molitor. *J. Biochem.* 116, 53–58. doi:10.1093/oxfordjournals.jbchem.a124502.

Moore, A. J., Beazley, W. D., Bibby, M. C., and Devine, D. A. (1996). Antimicrobial activity of cecropins. *J. Antimicrob. Chemother.* 37, 1077–1089. doi:10.1093/jac/37.6.1077.

Nayak, T., Mandal, S. M., Neog, K., and Ghosh, A. K. (2018). Characterization of a Gloverin-Like Antimicrobial Peptide Isolated from Muga Silkworm, Antheraea assamensis. *Int. J. Pept. Res. Ther.* 24, 337–346. doi:10.1007/s10989-017-9618-0.

Nie, Y., Zeng, X. C., Yang, Y., Luo, F., Luo, X., Wu, S., et al. (2012). A novel class of antimicrobial peptides from the scorpion Heterometrus spinifer. *Peptides* 38, 389–394. doi:10.1016/j.peptides.2012.09.012.

Okada, M., and Natori, S. (1985). Primary Structure of Sarcotoxin I, an Antibacterial Protein Induced in the Hemolymph of Sarcophaga peregrina (Flesh Fly) Larvae*. *J. Biol. Chem.* 260, 7174–7177.

Orivel, J., Redeker, V., Le Caer, J. P., Krier, F., Revol-Junelles, A. M., Longeon, A., et al. (2001). Ponericins, New Antibacterial and Insecticidal Peptides from the Venom of the Ant Pachycondyla goeldii. *J. Biol. Chem.* 276, 17823–17829. doi:10.1074/jbc.M100216200.

Park, S.-I., and Yoe, S. M. (2017). A novel cecropin-like peptide from black soldier fly, *Hermetia illucens* : Isolation, structural and functional characterization. *Entomol. Res.* 47, 115–124. doi:10.1111/1748-5967.12226.

Park, S. I., Kim, J. W., and Yoe, S. M. (2015). Purification and characterization of a novel antibacterial peptide from black soldier fly (Hermetia illucens) larvae. *Dev. Comp. Immunol.* 52, 98–106. doi:10.1016/j.dci.2015.04.018.

Rabel, D., Charlet, M., Ehret-Sabatier, L., Cavicchioli, L., Cudic, M., Otvos, L., et al. (2004). Primary Structure and in Vitro Antibacterial Properties of the Drosophila melanogaster Attacin C Pro-domain. *J. Biol. Chem.* 279, 14853–14859. doi:10.1074/jbc.M313608200.

Rahnamaeian, M., Cytryńska, M., Zdybicka-Barabas, A., and Vilcinskas, A. (2016). The functional interaction between abaecin and pore-forming peptides indicates a general mechanism of antibacterial potentiation. *Peptides* 78, 17–23. doi:10.1016/j.peptides.2016.01.016.

Ramírez-Carreto, S., Jiménez-Vargas, J. M., Rivas-Santiago, B., Corzo, G., Possani, L. D., Becerril, B., et al. (2015). Peptides from the scorpion Vaejovis punctatus with broad antimicrobial activity. *Peptides* 73, 51–59. doi:10.1016/j.peptides.2015.08.014.

Rodríguez, A., Villegas, E., Montoya-Rosales, A., Rivas-Santiago, B., and Corzo, G. (2014). Characterization of Antibacterial and Hemolytic Activity of Synthetic Pandinin 2 Variants and Their Inhibition against Mycobacterium tuberculosis. *PLoS One* 9, e101742. doi:10.1371/journal.pone.0101742.

Sagisaka, A., Miyanoshita, A., Ishibashi, J., and Yamakawa, M. (2001). Purification, characterization and gene expression of a glycine and proline-rich antibacterial protein family from larvae of a beetle, Allomyrina dichotoma. *Insect Mol. Biol.* 10, 293–302. doi:10.1046/j.0962-1075.2001.00261.x.

Saito, A., Ueda, K., Imamura, M., Atsumi, S., Tabunoki, H., Miura, N., et al. (2005). Purification and cDNA cloning of a cecropin from the longicorn beetle, Acalolepta luxuriosa. *Comp. Biochem. Physiol. - B Biochem. Mol. Biol.* 142, 317–323. doi:10.1016/j.cbpb.2005.08.001.

Segovia, L. J., Téllez Ramírez, G. A., Henao Arias, D. C., Rivera Duran, J. D., Bedoya, J. P., and Castaño Osorio, J. C. (2017). Identification and characterization of novel cecropins from the Oxysternon conspicillatum neotropic dung beetle. *PLoS One* 12, e0187914. doi:10.1371/journal.pone.0187914.

Téllez, G. A., and Castaño-Osorio, J. C. (2014). Expression and purification of an active cecropin-like recombinant protein against multidrug resistance Escherichia coli. *Protein Expr. Purif.* 100, 48–53. doi:10.1016/j.pep.2014.05.004.

Ursic-Bedoya, R., Buchhop, J., Joy, J. B., Durvasula, R., and Lowenberger, C. (2011). Prolixicin: A novel antimicrobial peptide isolated from Rhodnius prolixus with differential activity against bacteria and Trypanosoma cruzi. *Insect Mol. Biol.* 20, 775–786. doi:10.1111/j.1365-2583.2011.01107.x.

Vizioli, J., Richman, A. M., Uttenweiler-Joseph, S., Blass, C., and Bulet, P. (2001). The defensin peptide of the malaria vector mosquito anopheles gambiae: Antimicrobial activities and expression in adult mosquitoes. *Insect Biochem. Mol. Biol.* 31, 241–248. doi:10.1016/S0965-1748(00)00143-0.

Wu, S., Nie, Y., Zeng, X. C., Cao, H., Zhang, L., Zhou, L., et al. (2014). Genomic and functional characterization of three new venom peptides from the scorpion Heterometrus spinifer. *Peptides* 53, 30–41. doi:10.1016/j.peptides.2013.12.012.

Xie, L., Chen, G., Zhu, C., Zhu, B., and Hu, Y. (2009). Structural modification and expression of attacin a from glossina morsitans morsitans in E. coli and its antibacterial activities. *Int. J. Pept. Res. Ther.* 15, 255–261. doi:10.1007/s10989-009-9186-z.

Yamada, K., and Natori, S. (1993). Purification, sequence and antibacterial activity of two novel sapecin homologues from Sarcophaga embryonic cells: Similarity of sapecin B to charybdotoxin. *Biochem. J.* 291, 275–279. doi:10.1042/bj2910275.

Yi, H. Y., Chowdhury, M., Huang, Y. D., and Yu, X. Q. (2014). Insect antimicrobial peptides and their applications. *Appl. Microbiol. Biotechnol.* 98, 5807–5822. doi:10.1007/s00253-014-5792-6.

Yoe, S. M., Kang, C. S., Han, S. S., and Bang, I. S. (2006). Characterization and cDNA cloning of hinnavin II, a cecropin family antibacterial peptide from the cabbage butterfly, Artogeia rapae. *Comp. Biochem. Physiol. - B Biochem. Mol. Biol.* 144, 199–205. doi:10.1016/j.cbpb.2006.02.010.

Yoon, H. S., Lee, C. S., Lee, S. Y., Choi, C. S., Lee, I. H., Yeo, S. M., et al. (2003). Purification and cDNA cloning of inducible antibacterial peptides fromProtaetia brevitarsis (Coleoptera). *Arch. Insect Biochem. Physiol.* 52, 92–103. doi:10.1002/arch.10072.

Zeng, X. C., Zhou, L., Shi, W., Luo, X., Zhang, L., Nie, Y., et al. (2013). Three new antimicrobial peptides from the scorpion Pandinus imperator. *Peptides* 45, 28–34. doi:10.1016/j.peptides.2013.03.026.

Zheng, Z., Tharmalingam, N., Liu, Q., Jayamani, E., Kim, W., Fuchs, B. B., et al. (2017). Synergistic efficacy of Aedes aegypti antimicrobial peptide cecropin A2 and tetracycline against Pseudomonas aeruginosa. *Antimicrob. Agents Chemother.* 61. doi:10.1128/AAC.00686-17.

**
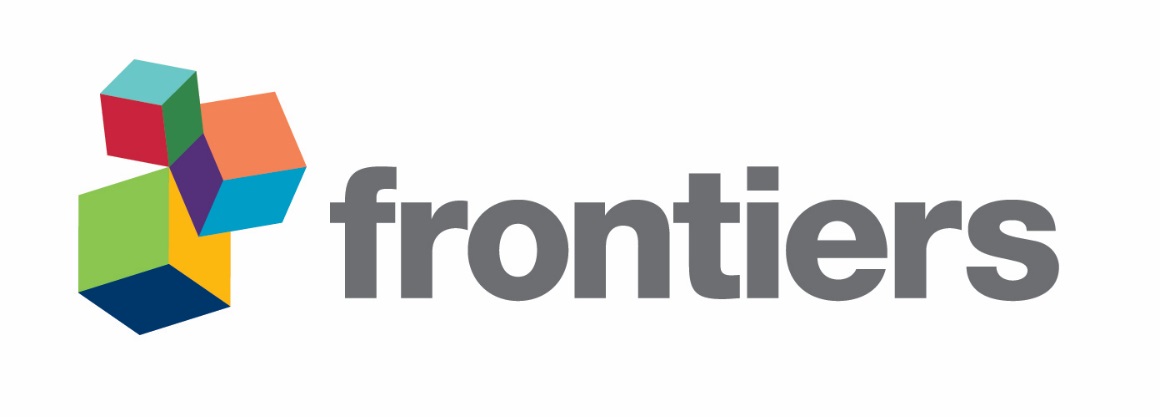
**
